# Immune priming in the insect gut: a dynamic response revealed by ultrastructural and transcriptomic changes

**DOI:** 10.1186/s12915-025-02334-4

**Published:** 2025-07-28

**Authors:** Moritz Baur, Nora K. E. Schulz, Lilo Greune, Zoe M. Länger, Jürgen Eirich, Iris Finkemeier, Robert Peuß, Petra Dersch, Joachim Kurtz

**Affiliations:** 1 Institute for Evolution and Biodiversity, University of Münster, Hüfferstrasse 1, 48149 Münster, Germany; 2 Center for Molecular Biology of Inflammation, University of Münster, Von-Esmarch-Strasse 56, 48149 Münster, Germany; 3 Institute of Plant Biology and Biotechnology, University of Münster, Schlossplatz 7, 48143 Münster, Germany; 4 Joint Institute for Individualization in a Changing Environment, University of Münster and Bielefeld University, North Rhine-Westphalia, Germany

**Keywords:** Innate immune memory, Evolution of immunity, Invertebrate, Insect, Pathogen, *Bacillus thuringiensis*, Virulence

## Abstract

**Background:**

Research on forms of memory in innate immune systems has recently gained momentum with the study of trained immunity in vertebrates and immune priming in invertebrates. Immune priming is an evolutionary ancient process that confers protection against previously encountered pathogens. However, despite the existence of immune priming across many invertebrate taxa, evolution and mechanisms of immune priming are still not well understood. Moreover, it is unclear how natural pathogens might elicit immune priming in their hosts.

**Results:**

Here we combine RNA sequencing with transmission electron microscopy to investigate the dynamic processes during priming in the gut of a well-established model for oral immune priming, consisting of the host *Tribolium castaneum* and its natural pathogen *Bacillus thuringiensis tenebrionis (Btt)*. We show that priming with specific, pathogen-derived virulence-relevant factors induces gut damage in *T. castaneum* larvae, triggering an early physiological stress response and upregulation of a distinct set of immune genes. This response diminishes over time yet enables the gut to upregulate genes known to interfere with *Btt* virulence when later exposed to infectious *Btt* spores.

**Conclusions:**

Our findings demonstrate that pathogen-derived factors inducing gut damage and stress responses prime gut tissue to provide more efficient protection against infection. These insights deepen our understanding of the mechanisms driving innate immune memory, which likely evolved as an adaptive response to natural pathogens.

**Supplementary Information:**

The online version contains supplementary material available at 10.1186/s12915-025-02334-4.

## Background

Immune systems are a universal feature across the tree of life. As checks and balance systems that discriminate between self and non-self, they allow for protection against harmful pathogens and parasites while limiting self-harm. Vertebrate animals possess both evolutionary ancient innate immunity and the more recent adaptive immune system, which provides specific memory. Adaptive immune systems evolved in cyclostomes and jawed vertebrates, whereas invertebrates lack an adaptive immune system in that sense [1, 2]. Over the past decades, the clear separation between innate and adaptive immunity has been challenged by work demonstrating forms of memory within innate immune systems in both vertebrates and invertebrates [3]. In vertebrates, innate immune memory that has been denoted “trained immunity” acts through the rewiring of metabolism and epigenetic imprinting of immune and non-immune (e.g., epithelial) cells [4–7].

Invertebrates have evolved multi-faceted mechanisms leading to enhanced survival of infections after a previous encounter with pathogens or their cues, despite the absence of the adaptive immune cellular machinery as found in vertebrates [8–13]. These processes, usually denoted as immune priming, are widespread across invertebrates and entail many distinct phenomena [14]. Experimentally, systemic or septic immune priming is achieved by pricking or injecting a host with sublethal doses, inactivated microbes, or immunoreactive cues derived from those microbes before infecting it, in the same way, with living microbes. This route of priming can be specific, providing a survival benefit only if the priming agent is the same as the infecting agent [8, 13, 15–17], or unspecific, providing a benefit during a subsequent infection even if the infecting agent is different from the one used for priming [11]. Immune priming is also possible via the oral route. Like septic immune priming, oral immune priming can be specific depending on the host and microorganism involved [8, 18, 19]. The two different routes of immune priming (septic and oral) likely function via different mechanisms [20], although it has also been reported in some organisms that oral immune priming can protect a host against a subsequent septic infection [21, 22]. Immune priming further is transmittable to subsequent generations, a phenomenon termed transgenerational immune priming [23–27]. While mechanisms of septic immune priming have been studied over the past years, similar work on oral immune priming is still scarce.

Immune priming via the oral route is likely a complex process. The microbiome seems to play a role in some species [16, 28, 29]. In mosquitoes and bean bugs, members of the microbiome take an active part by breaching the gut epithelium, thereby stimulating immune responses that provide systemic immunization [16, 29]. In an important model for oral immune priming, the red flour beetle *Tribolium castaneum*, a shift in the microbiome composition upon priming has been described [28]. Oral immune priming enables insects to better resist a subsequent infection by ingested pathogens in an environment with high pathogen exposure. When the priming agent is taken up via the oral route, the epithelium lining in the intestine is the first tissue reacting to the presented stimuli. An intriguing open question is whether pathogen virulence factors acquired through oral uptake interact with host processes to establish a primed state, particularly in systems where host and pathogen have likely co-evolved.

We here focus on gut-associated processes of oral immune priming in *T. castaneum* when confronted with its natural entomopathogen *Bacillus thuringiensis tenebrionis* (*Btt*). *B. thuringiensis* is a spore-forming Gram-positive bacterium. During sporulation, it produces crystal toxins (Cry toxins), which become solubilized upon ingestion by the host and form pores in the gut epithelium [31, 32]. Upon germination, *B. thuringiensis* produces enzymes and toxins to further damage its hosts’ gut tissue, ultimately leading to the death of the host [31, 32]. Once *B. thuringiensis* has exhausted the hosts’ nutrients, vegetative cells sporulate, ready to infect the next host [31, 32]. Greenwood et al. [33] found that oral immune priming of *T. castaneum* larvae with filter sterilized medium derived from sporulating *Btt* cells leads to a shift in whole-body gene expression profiles, which is still detectable 4 days later. Several immune genes remain upregulated in primed hosts upon pathogen exposure, and some genes are distinctly induced by challenge in those animals that only received a prior priming [33]. Moreover, recent work comparing proteomes of filtered growth media supernatants derived from closely related *B. thuringiensis* isolates, that either do or do not lead to priming of *T. castaneum* larvae, enabled the identification of candidate virulence factors that might induce oral immune priming, including the plasmid-encoded Cry3Aa toxin [34].

To shed light on the interplay of pathogen-derived factors with the host, we studied dynamic changes in the gut of *T. castaneum* larvae. As a result, we provide important insights into mechanisms that might constitute an evolutionarily ancient innate immune memory in invertebrates, in interplay with their natural pathogens.

## Results

### *T. castaneum* oral immune priming response in the gut peaks after 24 h and resembles an infected state

**Fig. 1:**
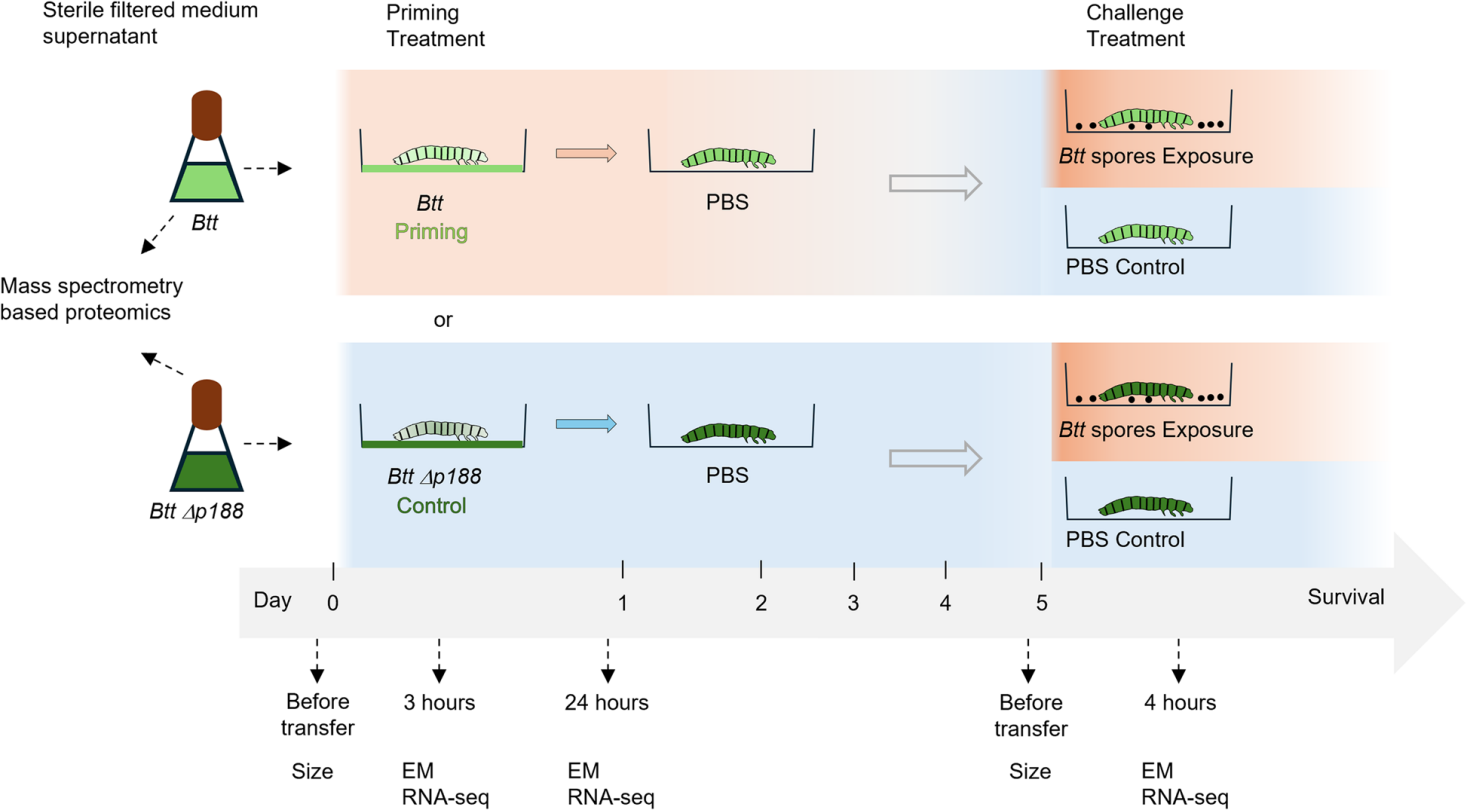
Study design. Before exposure to either priming- (*Btt*) or control (*Btt Δp188*) supernatant flour mixes (Priming treatment), we measured the size of 14-day-old larvae. Early time-point gut samples were taken at 3 h and 24 h post-exposure for electron microscopy (EM) and RNA-sequencing (RNA-seq). The remaining larvae were transferred onto PBS flour mixes after 24 h and left for four more days. Before transferring onto control (Phosphate buffered saline, PBS)- or exposure (*Btt* spores) flour mixes (Challenge treatment), we measured the size of the same larvae as in the beginning. Four hours after the transfer onto the challenge diets, gut samples were taken for both EM and RNA-seq again. The sterile-filtered spent culture media supernatants of *Btt* and *Btt Δp188* that were used for the Priming treatment were also compared regarding their proteome composition with LC MS/MS. For more details, please refer to the “Methods” section

We here used the *T. castaneum – B. thuringiensis tenebrionis* host–pathogen model to provide insights into the phenomenon of oral immune priming in the insect gut. We investigated ultrastructural changes using transmission electron microscopy together with changes in gene expression using RNA-seq of gut tissue of *T. castaneum* larvae at different time points after oral priming and infection. As an improved control for priming, we here used a *Btt* strain, which is cured of the Cry toxin-carrying plasmid (*Btt Δp188*) [34]. Because the only difference between the treatment and control *Btt* strain lies in the absence of this plasmid, their use enables us to directly identify bacterial candidate proteins responsible for priming (for the experimental design see Fig. 1).

**Fig. 2:**
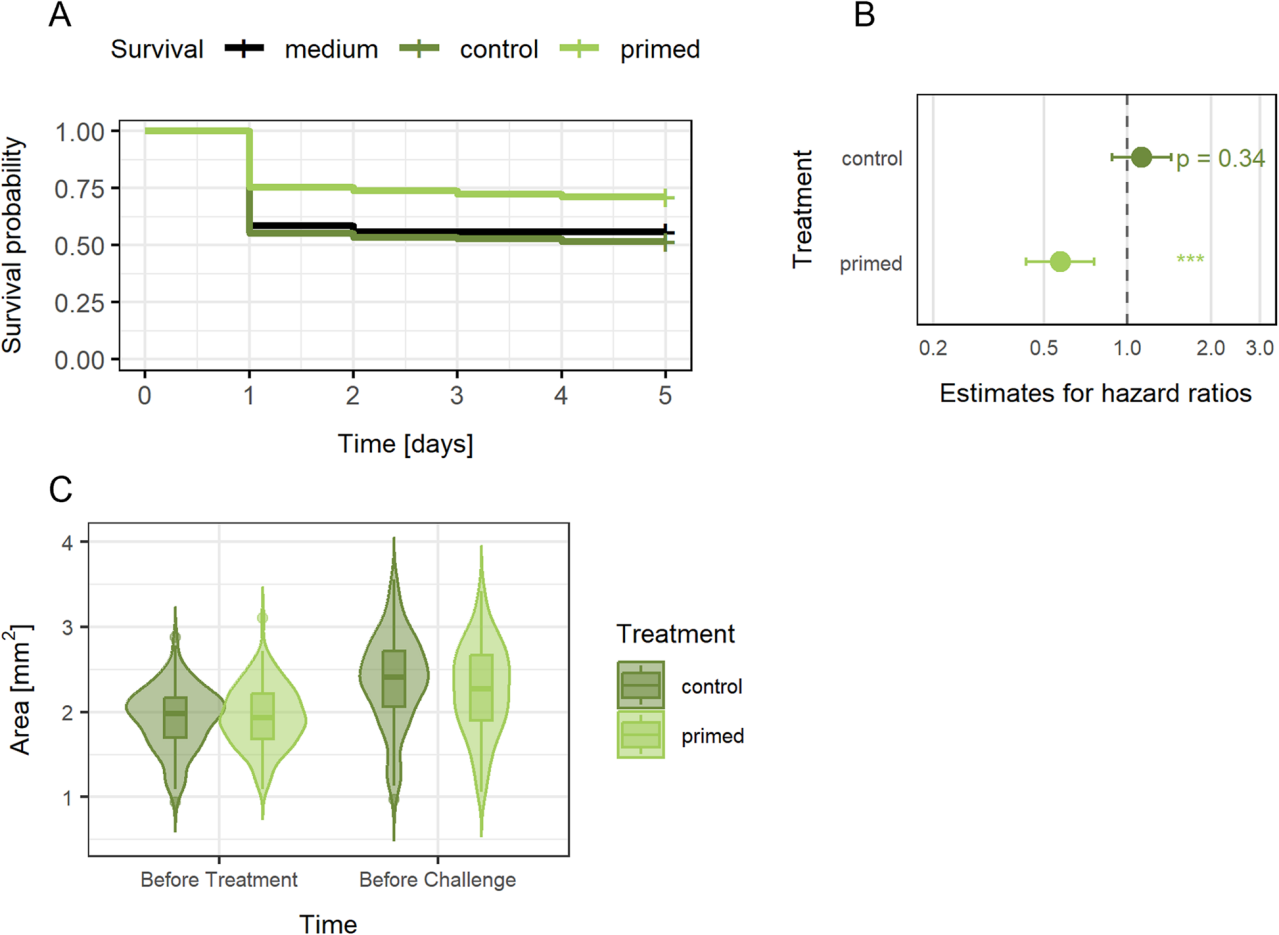
Infection survival and growth of *T. castaneum* larvae after priming.** A** Kaplan Meier curves displaying the survival of *T. castaneum* larvae exposed to either *Bt* medium diets (*n* = 495), control diets (*n* = 500), or priming diets (*n* = 501) upon exposure to infectious *Btt* spores. **B** Forest plot showing the treatment effect via the estimated hazard ratios of primed and control compared to medium control (black, dashed line at 1.0, *** = pvalue < 0.001). Hazard ratio ≥ 1 indicates less survival compared to medium control, hazard ratio ≤ 1 indicates enhanced survival. **C** Growth of *T. castaneum* larvae exposed to either priming (*n* = 132 larvae) or control (*n* = 135 larvae) diets. Larval size is given as their body area. The raw data for survival and growth can be found in Additional file 2: Table S1

The survival data derived from the present experiments confirm oral immune priming in *T. castaneum:* larvae showed improved survival after *Btt* infection following previous exposure to *Btt* culture supernatants (hazard ratio estimate = 0.57, *p* < 0.001), but not when exposed to *Btt Δp188* control supernatants (hazard ratio estimate = 1.13, *p* = 0.34) (each compared to *Bt* medium control, Fig. 2A,B, Additional file 1: Fig. S1). The efficacy of priming was 15.3% increased survival compared to the medium control and 19.7% compared to the *Btt Δp188* control. This increased survival probability after priming was accompanied by reduced growth in the treated individuals. During the time between priming and challenge exposure, larvae, which were initially of the same size (Fig. 2C, Estimate = 0.02038, *p* = 0.6586) grew significantly less if they received the priming treatment (16% ± SE 3.2%, *n* = 132) compared to the control (23.4% ± SE 3.6%, *n* = 135; estimate = − 0.13439, *p* < 0.001). While statistically robust, this difference reflects a relatively small effect size. Nonetheless such reduced growth can be an indicator of slower development to pupal and adult stages and therefore be of importance in holometabolous beetles [10].

**Fig. 3:**
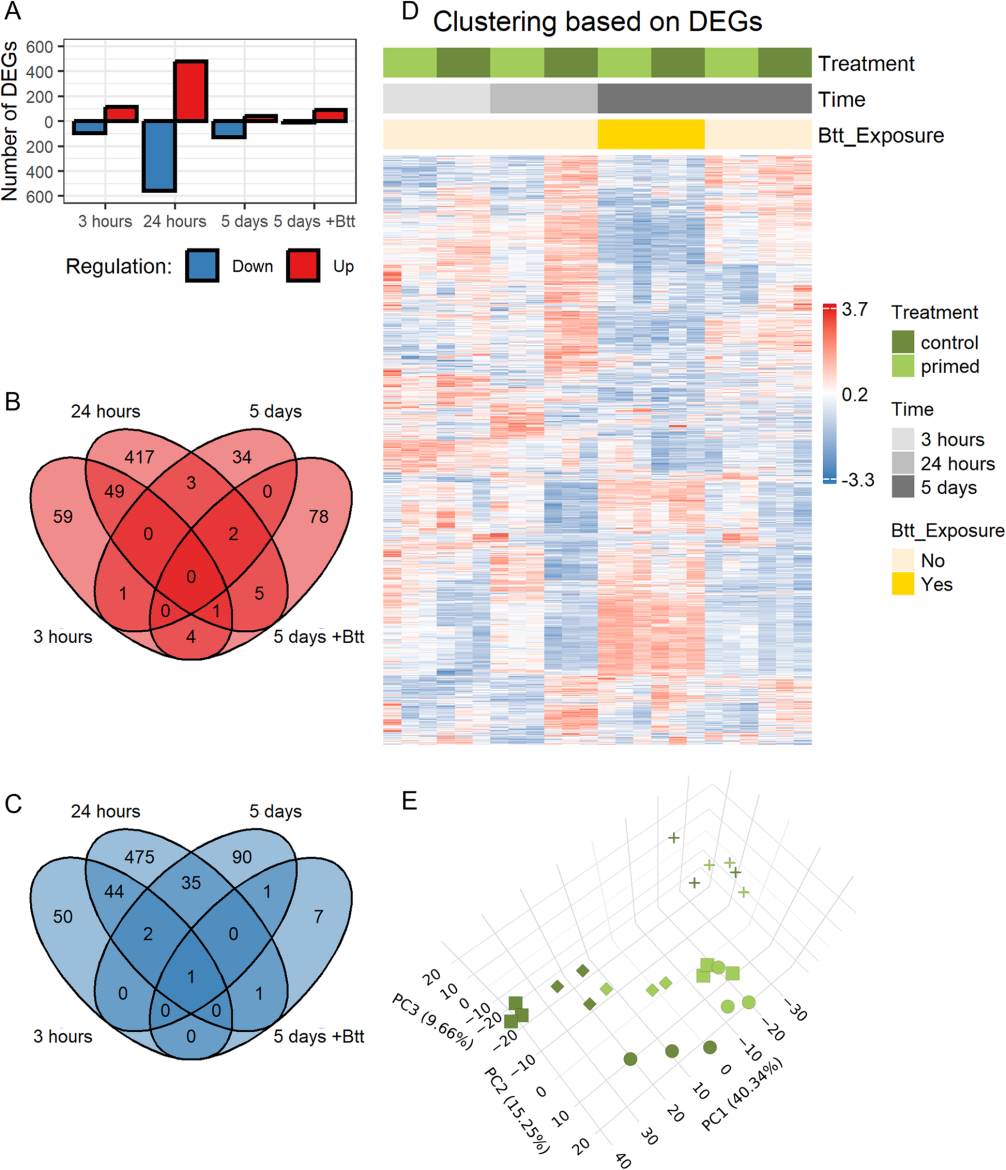
Overview of the results from the DESeq2 analysis.** A** Number of differentially expressed genes in guts from *T. castaneum* larvae exposed to priming *vs.* control diets (Down = lower expressed in primed guts, Up = higher expressed in primed guts), 3 h, 24 h, and 5 days after priming without and with challenge (+ *Btt*), analyzed with DESeq2 (Differentially expressed: Down in primed guts = Log2FC < − 0.5, FDR adjusted *p* < 0.05, Up in primed guts = Log2FC > 0.5, FDR adjusted *p* < 0.05). **B, C** Venn diagram for significantly up- (**B**) and downregulated (**C**) genes between primed and control larval gut tissues at the different timepoints (3 h, 24 h, 5 days, 5 days exposed to *Btt* spores). Overlapping areas represent shared DEGs between timepoints. The raw count data from the RNA-sequencing run can be found in Additional file 2: Table S2. **D** Heatmap representing the hierarchical clustering of the normalized gene expression for DEGs across all samples. Rows represent all DEGs identified in this study at all timepoints between primed and control larvae. Columns represent individual triplicate samples grouped by treatment, Time and exposure to infectious *Btt* spores (No = not exposed, Yes = exposed). Colors of the cells indicate the correlation of gene expression for a gene (row), in a sample (column). **E** Principal component analysis. 3-dimensional PCA for differentially expressed genes identified with DESeq2. Percentages represent the amount of variation explained by each principal component displayed. Circle = 3 hours, Square = 24 hours, Diamond = 5 days, Cross = 5 days +*Btt*

To analyze gene expression patterns in gut tissues from larvae that were either exposed to priming or control diets, we combined two approaches: DESeq2 was used to compare each gene’s expression between priming and control at each time point [35], and WGCNA was used to construct gene co-expression networks [36]. The number of differentially expressed genes (DEGs) between primed and control larval gut tissues varied over time (Fig. 3A), peaking at 24 h post-exposure with approximately 5–10 times as many DEGs compared to the other time points. In unchallenged larvae, more genes were downregulated in primed larval gut tissues after 5 days, whereas upon challenge with live *Btt* most DEGs were upregulated in primed larval gut tissues (Fig. 3A). When comparing the identity of DEGs between priming and control across the different time points, there was little overlap indicating that the priming response generally is dynamic (Fig. 3B, C). We also used the normalized gene expression of all DEGs identified between treatments across time points and performed hierarchical clustering, as well as principal component analysis (Fig. 3D, E). The samples clustered well by their group (time and treatment). Moreover, samples that were exposed to *Btt* spores (both primed and control) clustered together. The primed samples after three and 24 h clustered closest with one another and compared to the other samples closer to the *Btt* exposed samples, whereas the primed samples after 5 days clustered closer with the control samples. This shows that early after priming and exposure to *Btt* spores many genes are similarly strongly expressed.

**Fig. 4:**
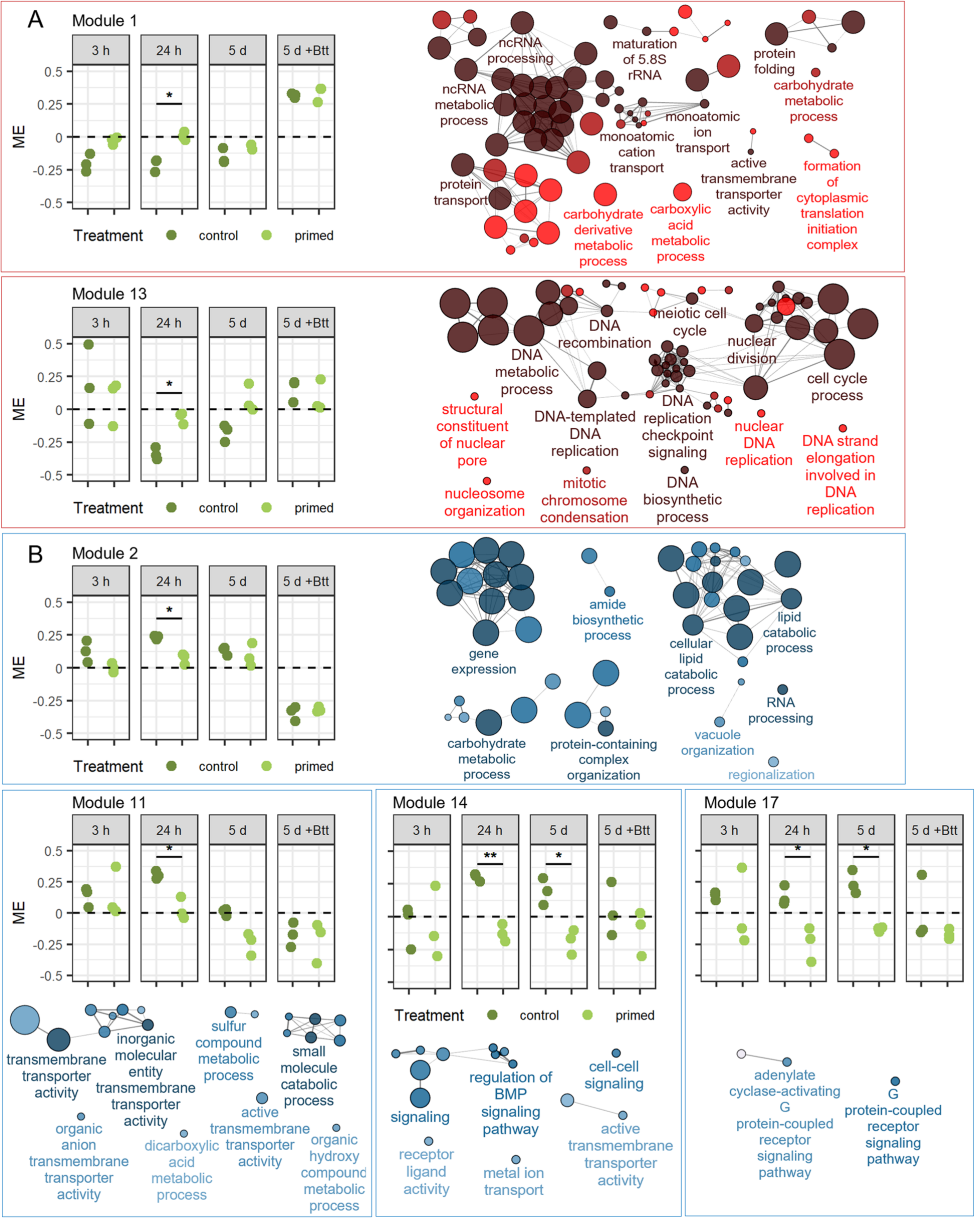
Significantly different WGCNA modules between larvae exposed to priming or control diets. The dot plots display module eigengene values (*y*-axis) vs samples grouped by timepoint (h = hours, d = days) and priming treatment (*x*-axis). Asterisks display FDR adjusted significance levels (* = *p* ≤ 0.05, ** = *p* ≤ 0.01). The networks represent Biological Processes contained in the modules identified with ClueGO. Only significantly enriched terms are included (Holm-Bonferroni adjusted *p* < 0.05). Each node represents a GO term. Node labels are based on the most significant GO term of interconnected GO terms. Node size represents the number of genes mapped to the node. Node color reflects significance level (Dark = highly significant, bright = less significant). Edges connect nodes based on shared genes (Kappa ≥ 0.4). **A** Modules 1 and 13 show higher module eigengene values for primed compared to control. **B** Modules 2, 11, 14, and 17 show lower module eigengene values for primed compared to control. WGCNA analysis is based on the data found in Additional file 2: Table S2 and Additional file 2: Table S3

After filtering out genes with low expression levels across samples, 10,814 genes of the total 17,052 genes remained for WGCNA. The WGCNA analysis identified 25 modules, with module 1 containing 2262 genes and module 25 containing 36 genes (Additional file 1: Fig. S2). Similarly, to the differential gene expression analysis, *Btt* exposed samples from control and priming treatment clustered well together upon Pearson correlation of the module eigengene values with the samples grouped by treatment and time (Additional file 1: Fig. S3). We then went on and statistically compared the module eigengene (ME) values at each timepoint between primed and control samples. Only the 24-h and 5-day time points displayed statistically different ME values. After 24 h, 7 modules displayed statistically different ME values (false discovery rate (FDR) adjusted *p* < 0.05) between primed and control samples (Modules 1, 2, 8, 11, 13, 14, 17, Fig. 4). Module 8 was excluded as no GO terms could be identified in this module. The gene ontology (GO) analysis of key modules revealed that biological processes associated with protein synthesis and folding are upregulated in primed larval guts after 24 h compared to controls, suggesting an increased cellular stress response (Fig. 4A, Module 1). Additionally, priming was associated with elevated expression of genes involved in cell cycle regulation and nuclear DNA-related processes, further indicating an increased stress state (Fig. 4A, Module 13). Conversely, Modules 2, 11, 14, and 17 comprised genes downregulated in primed larvae relative to controls (Fig. 4B). Processes such as lipid catabolism and gene expression were reduced, which, in conjunction with the upregulation of protein folding genes, may indicate endoplasmic reticulum (ER) stress. Downregulation of genes involved in cellular signaling, including the BMP signaling pathway, paired with upregulation of cell cycle genes, which might suggest shifts in cell differentiation. Notably, at the 5-day mark, Modules 14 and 17 also exhibited significant differences in module eigengene (ME) values between primed and control guts (Fig. 4B). These WGCNA findings underscore the potential for priming-induced stress, prompting us to combine transcriptomic insights with transmission electron microscopy (TEM) to investigate ultrastructural changes associated with gene expression dynamics in response to priming.

### The early priming response is characterized by damage-associated stress responses and an upregulation of immune genes

**Fig. 5:**
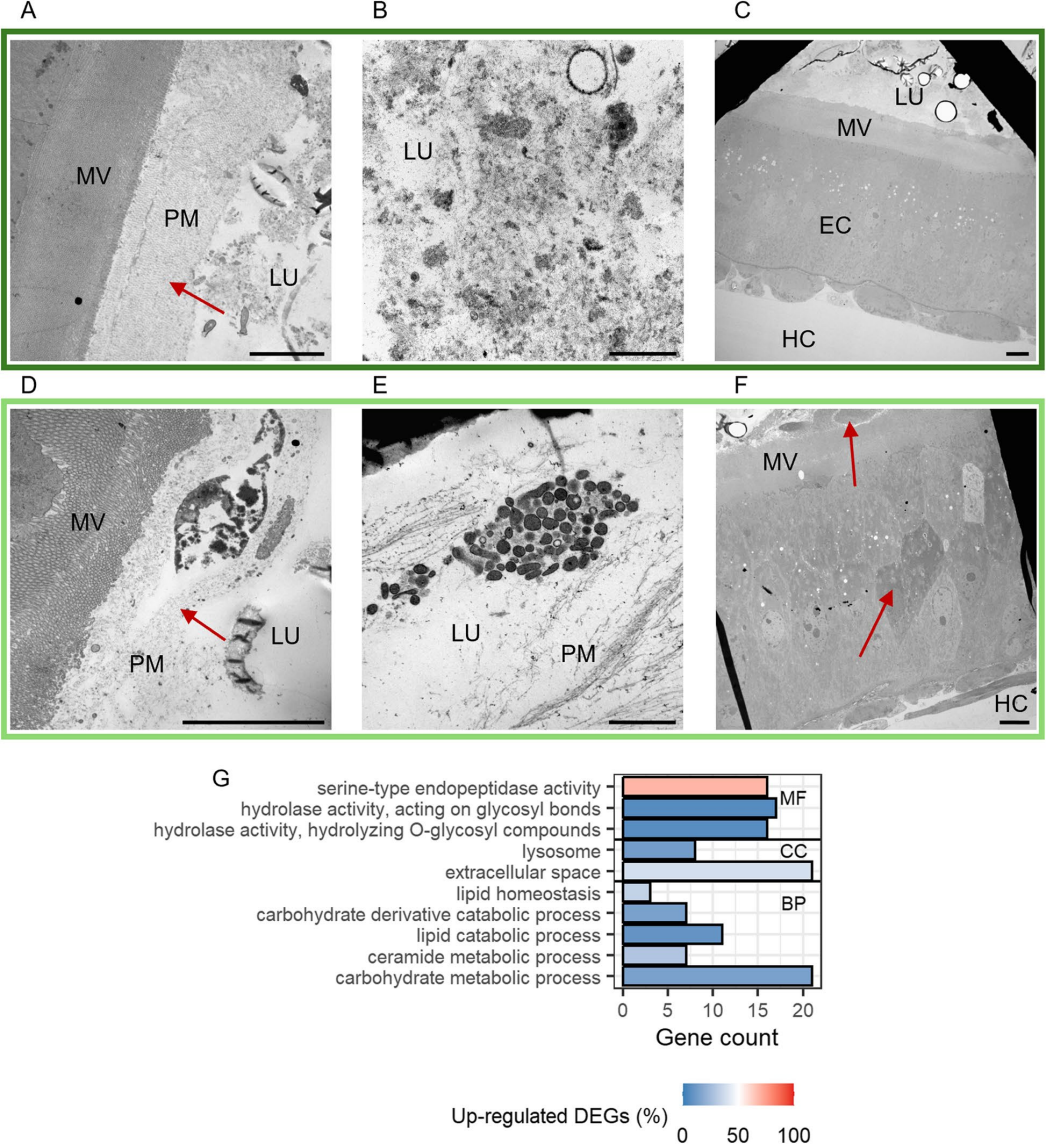
Differences between control (*Btt Δp188*) and priming (*Btt*) after 3 h.** A–F** Electron micrographs of control larvae (**A–C**) and primed larvae guts (**D–F**). Micrographs were chosen based on similar position and depth of sections between treatments. LU = gut lumen, MV = microvilli, PM = peritrophic matrix, EC = epithelial cell, HC = hemocoel. Red arrows: A, D = highlighting differences in peritrophic matrix, F = apoptotic cells. Scale bars: **A** = 5 µm, **B** = 1 µm, **C** = 5 µm, **D** = 5 µm, **E** = 1 µm, **F** = 5 µm. **G** GO term enrichment analysis for differentially expressed genes (DESeq2) between primed and control larval guts. Only GO terms with a significant enrichment are displayed (FDR adjusted *p* < 0.05). MF = Molecular Function, CC = Cellular Compartment, BP = Biological Process. The x-axis shows the number of genes that belong to a GO term, the color gradient indicates the ratio of down- (blue) and upregulated (red) genes identified with DESeq2

As early as 3 h after the exposure to the priming diets, the electron micrographs revealed a tattered peritrophic matrix (compare Fig. 5A and 5D, arrowheads) and a higher abundance of apoptotic cells in the gut of primed larvae (compare Fig. 5C and 5 F). We also observed an increase of membrane vesicles in the gut lumen after priming (compare Fig. 5B and 5E). Fitting these pathology observations in the gut of primed larvae, many DEGs at this time point are related to metabolic processes (Fig. 5G). Upregulated genes in primed larvae fell into the category of serine-type endopeptidases (Additional file 2: Table S4: 3 h: Category = “Proteases”), but also a ceramide synthase and genes encoding for proteins involved in intracellular protein degradation were significantly higher expressed (Additional file 2: Table S4: 3 h: Category = “Lipid metabolism” and “Proteases”). Most downregulated genes fell into categories of lipid and carbohydrate catabolic processes (Fig. 5G, Additional file 2: Table S4: 3 h: Category = “Lipid metabolism” and “Glycosidases”). None of the modules derived from the WGCNA analysis displayed significantly different ME values 3 h after priming.

**Fig. 6:**
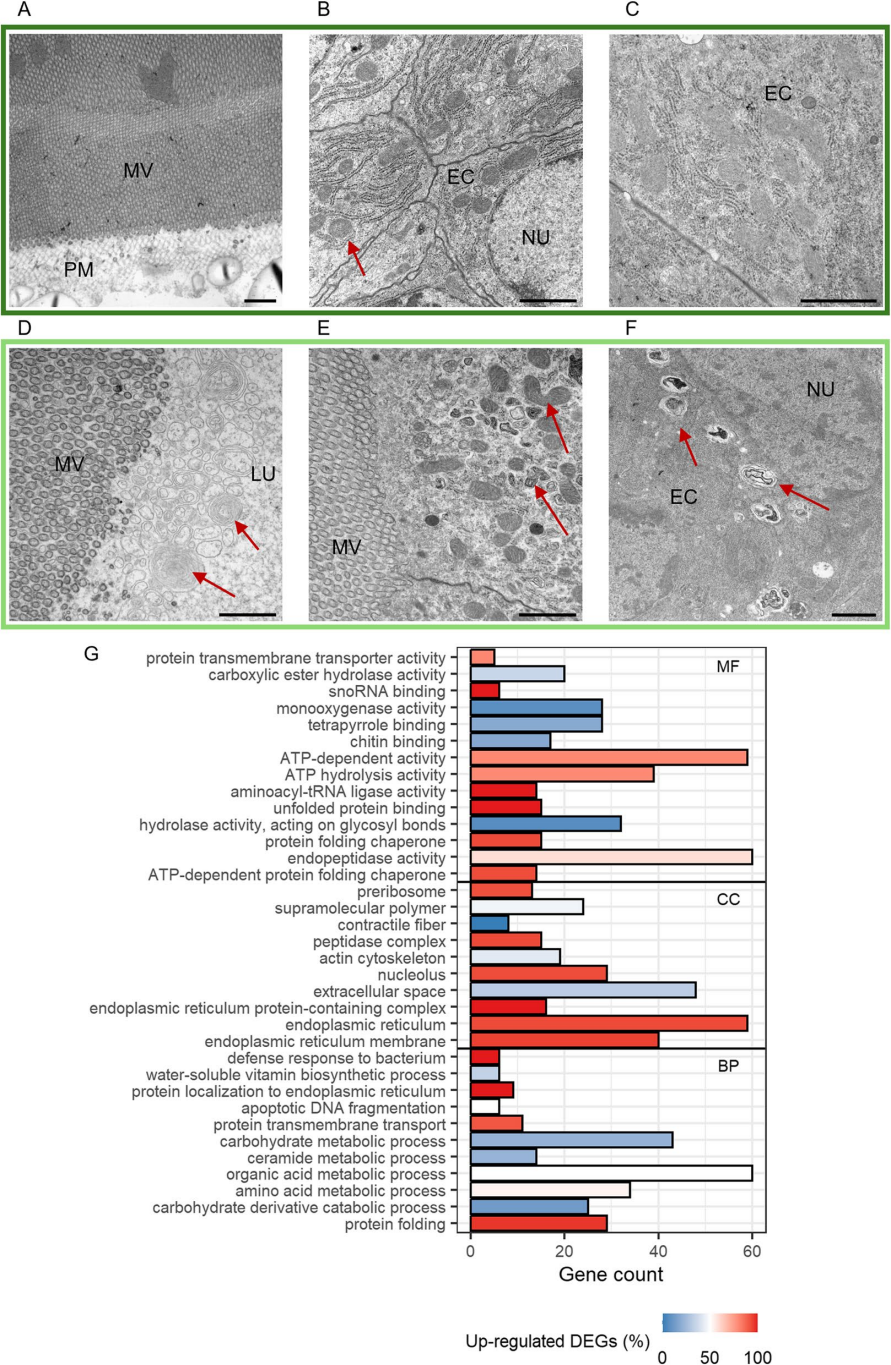
Differences between control (*Btt Δp188*) and priming (*Btt*) after 24 h.** A–F** Electron micrographs of control larvae (**A–C**) and primed larvae guts (**D–F**). Micrographs were chosen based on similar position and depth of sections between treatments. LU = gut lumen, MV = microvilli, PM = peritrophic matrix, EC = epithelial cell, NU = nucleus. Red arrows: A, D = “normal” (A) and “stressed” (D) mitochondria, E = autolysosomes, F = putative membrane residues originating from epithelial cells. Scale bars: **A–F** = 5 µm. **G** GO term enrichment analysis for differentially expressed genes (DESeq2) between primed and control larval gut tissues. For details, see Fig. 5

In line with the peak in the number of DEGs after 24 h, we detected stronger differences in the gut ultrastructure between primed and control larvae at this time point. In primed larvae, the tattered peritrophic matrix and increased apoptosis detected after 3 h persisted. Still, additional structures in the gut lumen and in between microvilli appeared, which seem to be membrane residues originating from gut epithelial cells (compare Fig. 6A and 6D, arrowheads). Both WGCNA and DESeq2 revealed downregulated genes in primed larval gut tissue that clustered into GO categories of lipid and carbohydrate catabolic processes (Fig. 4B: Module 2, Fig. 6G, Additional file 2: Table S4: 24 h: Category = “Lipid metabolism” and “Glycosidases”), which coincides with the disturbance of cellular membranes and the peritrophic matrix (PM) on the ultrastructure level. At the same time, genes encoding for chitin synthase and ceramide synthase were upregulated, possibly counteracting the degradation of cellular membranes and the PM. Also, mitochondria in the epithelial cells of primed larval guts showed signs of stress, such as elongated shapes and shrinkage (compare Fig. 6B and 6E, arrowheads). Additionally, we observed an increased number of autolysosomes, presumably due to the increased degeneration of mitochondria (compare Fig. 6C and 6F). This stress on the ultrastructure level within the epithelial cells was also reflected by a high number of genes that clustered into GO terms that involve the synthesis, modification, transport, and degradation of proteins, especially in the endoplasmic reticulum, which was again indicated by both DESeq2 and WGCNA (Fig. 4A: Module 1, Fig. 6G, Additional file 2: Table S4: 24 h: Category = “ER stress”). We also found the GO term “defense response to bacterium” to be significantly enriched in primed larval guts at this time point (Fig. 6G). Within this category are two gene copies of *Attacin*, *Coleoptericin*, and *Defensin*, respectively, as well as a gene encoding for an inducible metalloprotease inhibitor (Additional file 2: Table S4: 24 h: Category = “Immune”). Also, two genes encoding for peptidoglycan recognition receptors were differentially regulated. Further significant modules identified by WGCNA with higher gene expression profiles in primed larval gut tissues contained genes that participate in the recombination, replication, and repair of DNA and genes associated with cell cycle processes in the nucleus (Fig. 4A: Module 13). Modules that were associated with a lower gene expression profile contained genes involved in transmembrane transporter activities and cell-to-cell signaling (Fig. 4B: Modules 11, 14, and 17).

#### Reduced gene expression differences after 5 days, while signs of stress at the ultrastructural level persist

**Fig. 7:**
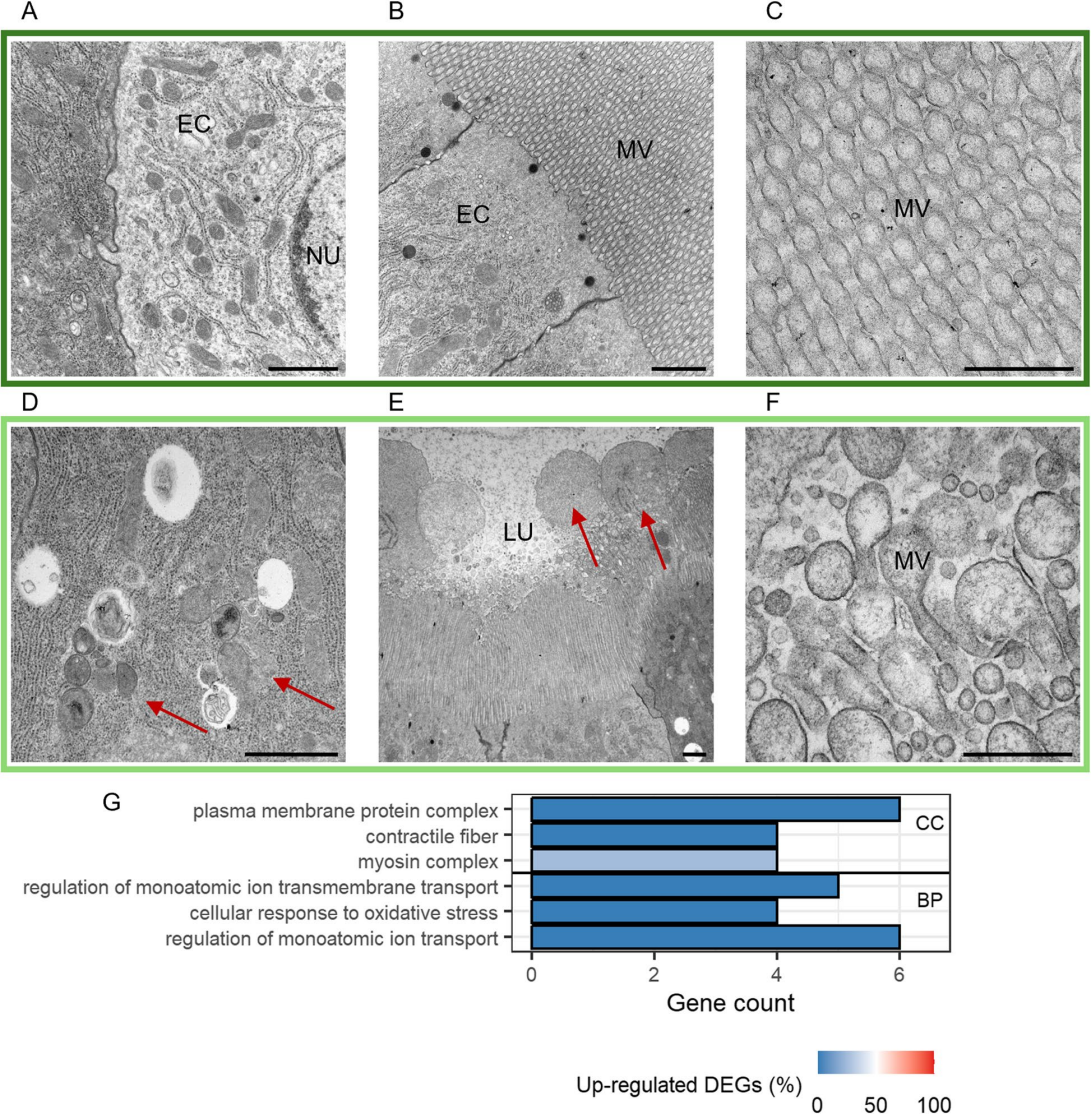
Differences between control (*Btt Δp188*) and priming (*Btt*) after 5 days. **A–F** Electron micrographs of control larvae (**A–C**) and primed larvae guts (**D–F**). Micrographs were chosen based on similar position and depth of sections between treatments. LU = gut lumen, MV = microvilli, EC = epithelial cell, NU = nucleus. Red arrows: D = stressed endoplasmic reticulum, E = swollen microvilli. Scale bars: A = 1 µm, B = 1 µm, C = 500 nm, D = 1 µm, E = 1 µm, F = 500 nm. **G** GO term enrichment analysis for differentially expressed genes (DESeq2) between primed and control larval gut tissues. For details, see Fig. 5

After 5 days, we still observed structural differences in the gut of primed larvae, such as the abovementioned unusual shapes of mitochondria or the increased abundance of autolysosomes (compare Fig. 7A and 7D). Additionally, we detected the occurrence of signs of stress in the ER of epithelial cells, namely the degeneration of ribosomes and even the degradation of the ER in primed larvae (Fig. 7D). The most prominent change, however, was the microvilli (MV) swelling in primed larval guts (compare Fig. 7B, E, C, F). These swellings occurred towards the tip of the MV and finally led to the extrusion of MV debris. Genes that were downregulated in primed larvae at this time point encoded, among others, for proteins that are part of the cytoskeletal architecture or are involved in detoxification processes (Fig. 7G, Additional file 2: Table S4: 5d: Category = “Cytoskeletal”, “Monooxygenases”). After 24 h, genes encoding for transmembrane transporter activity were downregulated as well as revealed by WGCNA and DESeq2 (Fig. 4B: Modules 14 and 17, Fig. 7G, Additional file 2: Table S4: 5d: Category = “Transporters”).

##### Upon exposure to live Btt, primed guts show higher expression of genes involved in detoxification processes

**Fig. 8:**
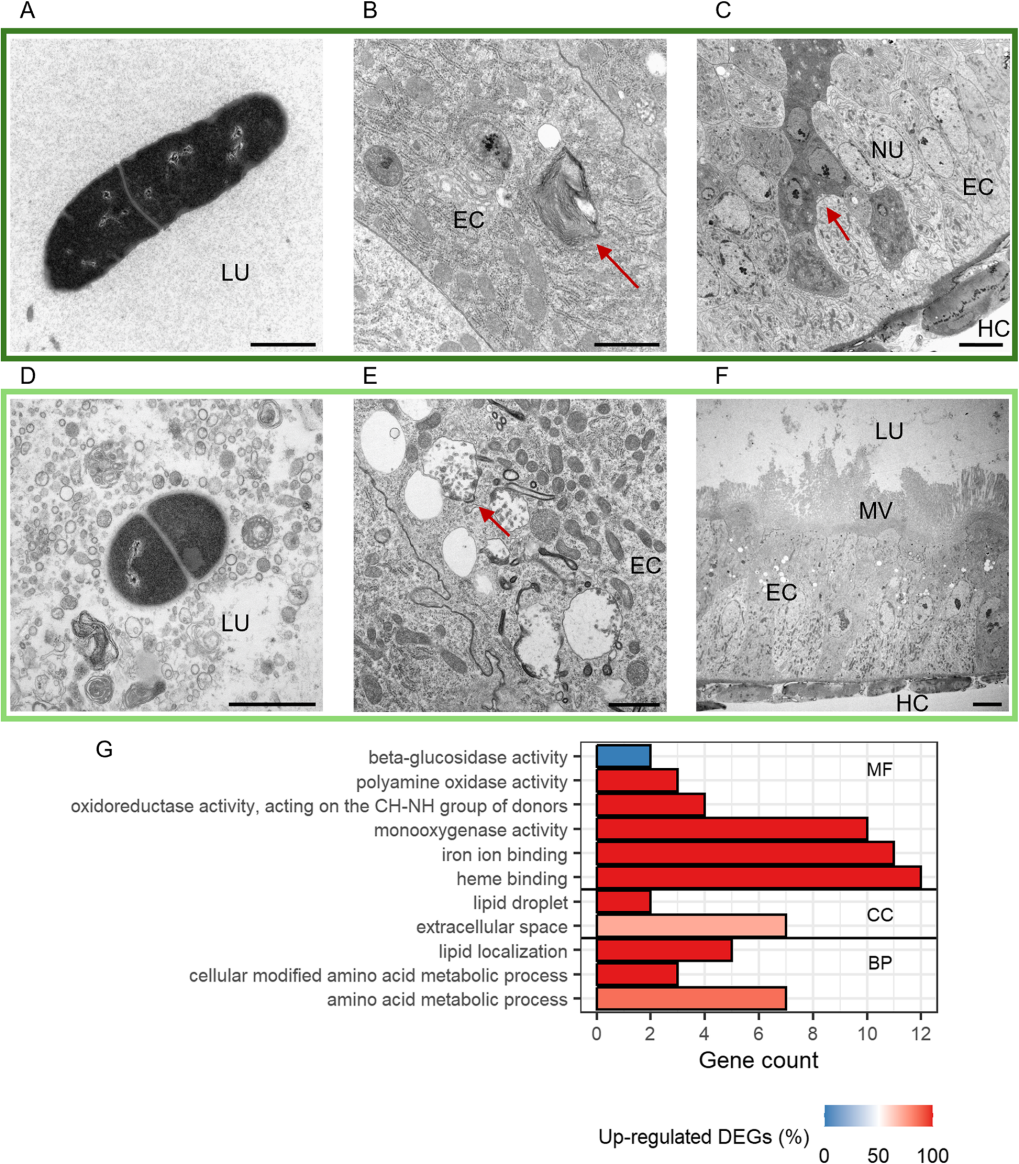
Differences between control (*Btt Δp188*) and priming (*Btt*) after 5 days and 4 h after exposure to infectious *Btt*.** A–F** Electron micrographs of control larvae (**A–C**) and primed larvae guts (**D–F**). Micrographs were chosen based on similar position and depth of sections between treatments. LU = gut lumen, MV = microvilli, EC = epithelial cell, NU = nucleus, HC = hemocoel. Red arrows: B = lysosomal structure, C = apoptotic cells, E = intracellular vesicles. Scale bars: A = 1 µm, B = 1 µm, C = 5 µm, D = 1 µm, E = 1 µm, F = 5 µm. **G** GO term enrichment analysis for differentially expressed genes (DESeq2) between primed and control larval gut tissues. For details, see Fig. 5

Five days after exposure to the priming or control diets, the guts of larvae that were exposed to *Btt* spores showed signs of infection in all samples. Vegetative *Btt* cells (Fig. 8A and 8D), lysosomal structures (Fig. 8B), and intracellular vesicles (Fig. 8E) were present in both control and primed larvae. In some gut sections, we detected an increased number of apoptotic cells (Fig. 8C), whereas other sections displayed a stressed MV rim (Fig. 8F). Primed and control larval guts hardly differed, as the biggest influence on gut integrity seemed to be the number of germinated *Btt* cells, which varied largely even within the treatment. In line with this, the number of DEGs between primed and control larvae was the lowest among the investigated time points (Fig. 3A). The only significant GO term containing downregulated genes was “beta-glucosidase activity”, including an acidic chitinase (Fig. 8G). Genes that were upregulated in primed larvae upon *Btt* exposure encoded for detoxification enzymes, which were downregulated in non-exposed larvae at this timepoint, lipid binding proteins and proteins containing leucine-rich repeats (Fig. 8G, Additional file 2: Table S4: 5d_E: Category = “Detoxification”,”Lipid binding”, “LRR”). Similar to 3 h after priming, none of the modules derived from the WGCNA analysis displayed significantly different module eigengene values after the spore exposure treatment.

##### Priming-associated damage in the gut is likely caused by virulence factors present in the priming diet

**Fig. 9:**
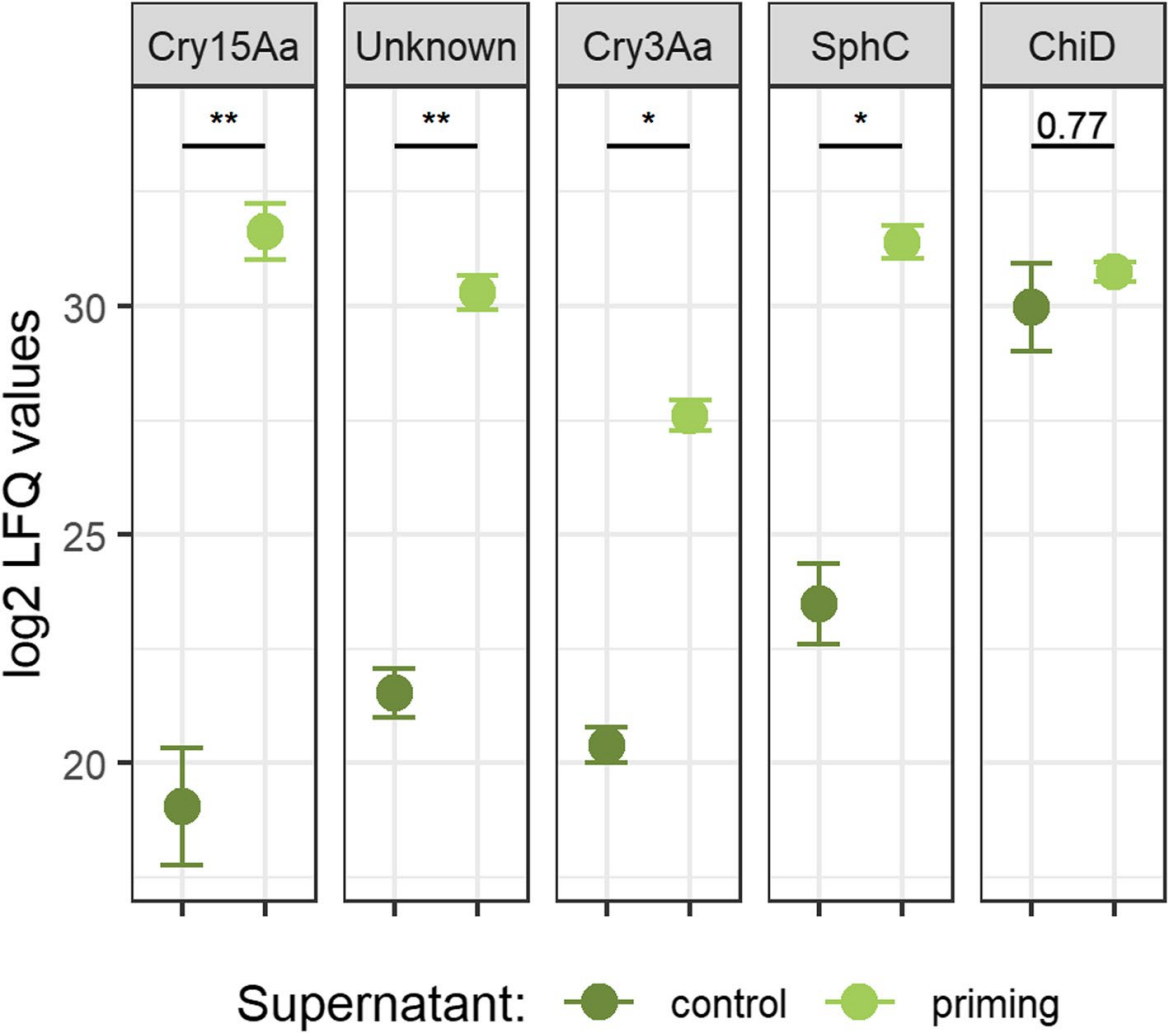
Exemplary proteome expression differences between priming (*Btt*) and control (*Btt Δp188*) spent culture media supernatants. Depicted are the mean values of the normalized and log2-transformed label-free quantification values after imputation for missing values (*n* = 4). Error bars represent the standard error. ** = *p* value < 0.01, * = *p* value < 0.05. SphC = Sphingomyelinase C, ChiD = Chitinase D

When we compared the proteomes of priming (*Btt*) and control (*Btt Δp188*) centrifuged, sterile filtered media supernatants, the only significantly differentially enriched protein group was a Sphingomyelinase C, which showed increased expression in the priming inducing *Btt* supernatant (Additional file 1: Fig. S4A). As expected, proteins that are encoded on the Cry-toxin carrying 188 kb plasmid were below detection limit in the *Btt Δp188* control supernatants, where this plasmid was removed. Among those proteins were the crystal toxins Cry3Aa and Cry15Aa (Additional file 2: Table S5A). Sphingomyelinase C is encoded on the same plasmid. The low expression level detected in 3 of 4 control media might be explained by another homologous Sphingomyelinase encoded on the chromosome and containing identical peptide fragments. Upon imputation of missing values, Cry15A, Cry3Aa, and Sphingomyelinase C were among the few protein groups that were significantly higher expressed in priming spent culture media supernatants (Fig. 9, Additional file 1: Fig. S4B, Additional file 2: Table S5B and S5C). Another virulence factor, Chitinase D, that was previously found to be differentially expressed in *Btt* compared to another *Bt* strain, was highly expressed in both control and priming supernatants [32].

## Discussion

**Fig. 10:**
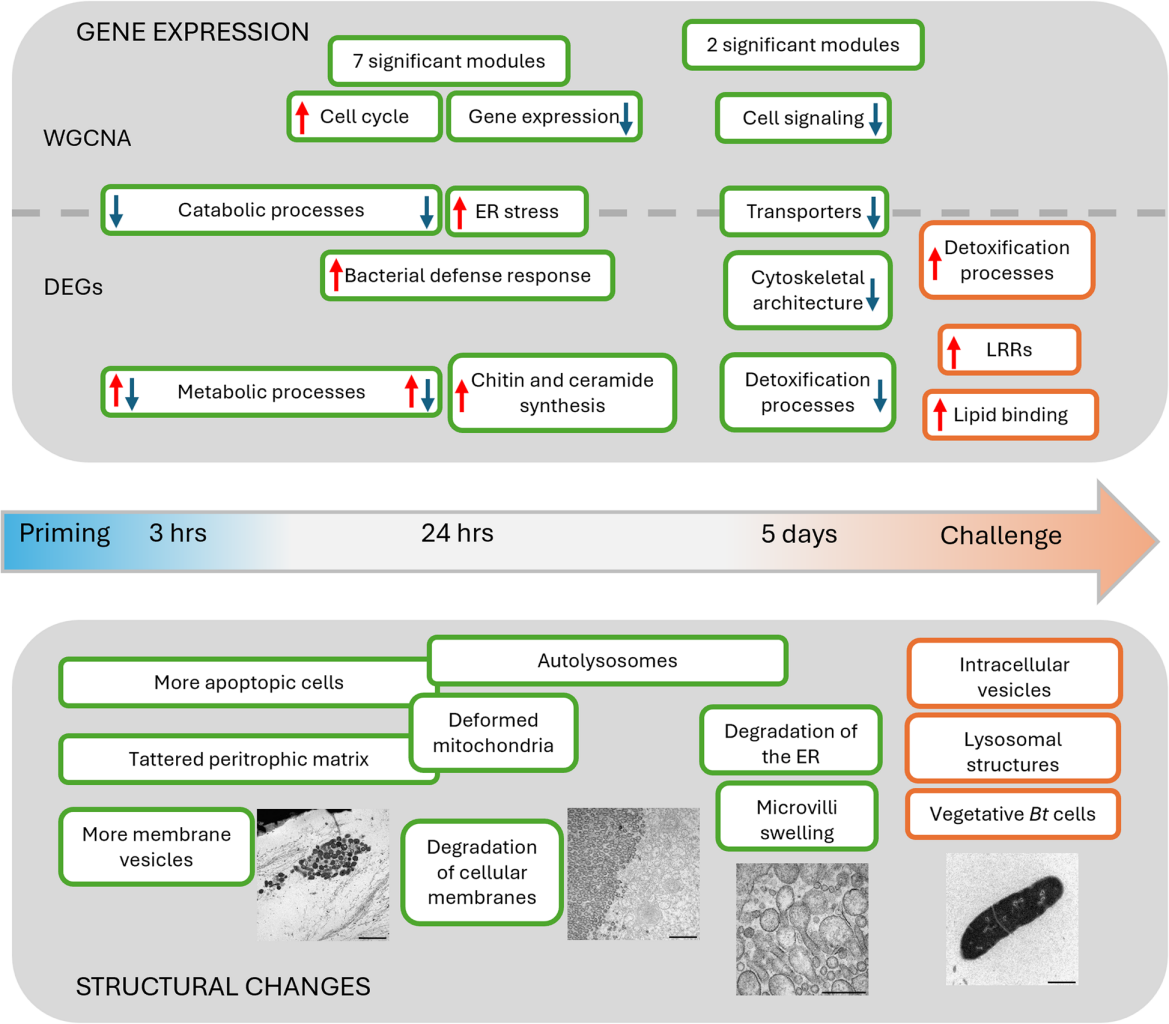
Results overview. The upper panel displays differences in gene expression between primed vs control larval guts with regard to both WGCNA and differential gene expression analysis (DESeq2). The lower panel describes changes on the ultrastructural level in primed compared to control guts. The arrow indicates the timepoint after priming treatment as well as after challenge with live *Btt* spores

Immune priming is the ability of evolutionary ancient innate immune systems to provide protection against previously encountered pathogens. To gain insights into the dynamic responses following oral immune priming in the insect gut, we combined electron microscopy and RNA-sequencing of *T. castaneum* larval guts after priming and upon exposure to live *Btt* spores. Our results show a strong reaction in the gut associated with *Btt*-triggered tissue damage early after priming, including the upregulation of immune genes, that diminishes over time but enables a targeted response upon re-encountering infective *Btt* spores (for an overview of the results see Fig. 10). Primed larvae survive a *Btt* infection better, but grow significantly less, which in holometabolous insects can lead to later pupation and smaller adults [10]. These factors negatively affect fitness and show that priming is a costly phenomenon that can come at a trade-off.

Priming immediately induced strong damage in the gut epithelium and this damage became more pronounced after 24 h. Similar responses to the here observed membrane and micro-vesicle shedding have been reported in HeLa cell cultures and mice upon exposure to pore-forming toxins and seem to aid in the repair of damaged cellular membranes [37, 38]. Overall, the observed changes in gut ultrastructure can likely be attributed to the presence of virulence factors in the priming diet and resemble effects of natural *Bt* infections [31, 32]. Chitinases break down the peritrophic matrix, and Cry toxins, activated by midgut proteases, bind to receptors on epithelial cells, ultimately leading to cell death [31, 39, 40]. Sphingomyelinases further damage cellular membranes by hydrolyzing sphingomyelin [41, 42]. After 24 h in accordance with the increase of signs of damage, the number of differentially expressed genes (DEGs) between treatments also reached its peak. Among the DEGs were cysteine- and serine proteases, the main digestive enzymes in *T. castaneum* [43], and similar shifts in expression in response to Cry toxin exposure have been observed in *Tenebrio molitor* [44]. Also, other studies indicate an influence of the gut enzyme composition on the Cry3Aa toxin activity [45, 46]. Additionally, after 24 h, two serine protease inhibitors (serpins), which have been linked to insect immunity were upregulated, suggesting active regulation of these proteases [47]. Many genes encoding for glycosyl hydrolases, enzymes involved in the degradation of chitin, were downregulated after 24 h. This, together with the upregulation of a chitin synthase, could indicate a strategy to suppress chitin catabolism in the presence of externally induced peritrophic matrix damage. Genes encoding for ceramidases and sphingomyelinases, enzymes involved in the degradation of membrane-associated fatty acids, were mostly downregulated. In mammalian cells such downregulation aids the regeneration upon exposure to pore-forming toxins [48]. Sphingomyelins and ceramides not only are major constituents of cellular membranes but also play roles in endoplasmic reticulum (ER) homeostasis and immune signaling [49–52]. The presence of sphingomyelinase C the in priming-inducing supernatant, together with the differential expression of sphingomyelin-associated genes and the occurrence of membrane residues in the gut lumen after priming, suggests a disturbance of lipid homeostasis. In the intestines of cockroaches, ER stress and lipid homeostasis are connected, and their disturbance increases the expression of pro-inflammatory genes [53]. We also saw an upregulation of genes that are linked to such ER-associated stress, including genes encoding for membrane-spanning proteins in the ER [54–57] and components necessary for the correct degradation of proteins [58]. Also, genes involved in the unfolded protein response (UPR), a cellular stress response related to ER stress, were upregulated [59–63]. *Aedes aegypti* requires these genes to survive Cry11Aa toxin exposure [64].

Many of the upregulated cell cycle and checkpoint signaling genes in primed larvae have been reported to correlate with ER stress, and the influence of ER stress on DNA integrity, repair and epigenetic changes, is a topic of active research [65–67]. In *Drosophila*, pathogen exposure leads to divergent stem cell decision and a change in epithelial cell composition [68]. While genes involved in gene expression were downregulated in primed larvae, tRNA synthetase- and ribosome genes were upregulated, potentially reflecting an increased demand in protein synthesis while controlling for unbalanced gene expression [69]. Two genes encoding peptidoglycan recognition proteins (PGRP) that were differentially regulated after 24 h previously showed similar expression patterns following septic infections in *T. castaneum* with *Micrococcus luteus* [70]. Additionally, Gram-positive bacteria targeting antimicrobial peptides (AMPs) were upregulated after 24 h [71–73], indicating an immune response following priming. Interestingly, genes encoding for monooxygenases, that are known to be involved in detoxification processes and are overexpressed in some *Bt*-resistant insects [74, 75], were mostly downregulated at this time point. Overall, the differential gene expression pattern in the guts of primed larvae resembles that of whole larvae shortly after exposure to Gram-positive bacteria in other work [20, 33].

Five days after exposure, signs of stress remained visible in primed guts, e.g., enlarged microvilli (MV) heads. In rats, such MV “blebs” contain catalytically active enzymes with regulatory properties against microbes [76–78]. Future work could investigate whether these MV “blebs” provide the same function in the beetle gut. The gene expression profiles between treatments were more similar compared to the early time points. Most DEGS were downregulated in primed guts, including genes encoding for cytoskeletal proteins, which might correlate with the aberrant appearance of the MV [77]. Several transmembrane transporter genes, encoding for proteins that are similar to the reported putative functional receptors for Cry toxins in *T. castaneum* were downregulated as well [79–81]. Similar to 24 h, genes encoding for monooxygenases were mostly downregulated indicating strict regulation [82].

After exposure to infectious *Btt* spores, vegetative *Btt* cells were visible in the gut and the epithelium showed severe signs of damage in both control and primed larvae. Overall, the effects of the infection seem so strong that they override smaller, priming-associated differences at the ultrastructural level. Coinciding with this, the number of DEGs between primed and control guts was the lowest upon exposure. Most of these DEGs were upregulated in primed guts and contain pathogen-binding and immune signaling molecules such as apolipophorinI/II, leucine-rich repeats containing proteins, odorant binding receptors, and a scavenger receptor [83–88]. Previous studies found similar upregulation patterns for some of these genes in the same model system [33, 89]. The second highest upregulated gene in primed larvae was a gene encoding for Arylphorin, a mitogen in a Lepidopteran host that has been associated with *Bt* resistance [90, 91]. Genes encoding for enzymes involved in detoxification processes were upregulated upon *Btt* exposure, which was in contrast to after 24 h and after 5 days without exposure. In conclusion, the upregulation of binding and detoxification processes could imply a strategy to prevent damage imposed by the virulence factors of *Btt*.

For the first time, we described in detail the changes in the gut of *T. castaneum* larvae upon oral immune priming. In mosquitos and bean bugs, the microbiome breaches the epithelial cell lining, thereby influencing immune cell differentiation [16, 29]. Indeed, the microbiome in our system is needed for successful oral immune priming as well [28], and a change in its composition is described upon priming [30]. However, we here could not detect any signs of bacterial breaching in the gut epithelia on the ultrastructural level upon priming, and it is debated whether *Btt* breaches the gut before host death in *T. castaneum* [92]. Pinaud et al. [18] showed that in snails, oral immune priming leads to a shift from a cellular immune response upon the first encounter with the parasite to a humoral response after the second encounter. In their system, however, the *Schistosoma* parasite forms sporocysts that develop within the host tissue, representing a different pathogenesis than that of *Btt*. These works highlight the differences in the investigated host–parasite models and reflect the diversity of immune priming phenomena in invertebrates [93]. In our case, virulence factors of *Btt* induce a stress state in the gut of *T. castaneum* that resembles an actual infection at the ultrastructural and gene expression level. This stress state initially induces an immune response that diminishes over time, but upon re-infection with live *Btt* spores results in upregulation of protective genes against *Btt*. Greenwood et al. [33] found an increased immune response in primed *T. castaneum* larvae 6 h after exposure to life *Btt* using whole larvae. Thus, investigating the potential crosstalk between the gut epithelium and the hemocoelic side would be interesting. The identified changes in the gut might induce hemocyte migration or differentiation and fat body gene expression. This could lead to systemic immune responses and memory, which might aid protection against oral *Btt* infection and potentially also septic infection as in other systems [29]. An important aspect to mention is that we here only investigated a rather early timepoint after infection. Later sampling for example after 12 h could potentially reveal faster healing or differential regulation of immune genes in the gut tissue of primed larvae.

Our work sheds light on the principles of evolutionary ancient innate immune memory. With respect to the five immune memory criteria as worked out by Pradeu and Du Pasquier [94], immune priming in *T. castaneum* meets the following: it is specific to a certain degree both via the septic and oral route [15, 19], it might enable a faster response as indicated by the early upregulation of protective genes in the gut found in this study as well as the upregulation of immune genes at whole larval level found by Greenwood et al. [33]. Protection in orally primed larvae lasts for at least 5 days, as shown in this study, and up to 8 days, as in some septic studies, and can be transmitted to subsequent generations [16, 25]. The initial priming response in the gut diminishes the gene expression level over 5 days, indicating extinction.

But how could memory be formed in the insect gut epithelium? Work by Liu X et al. [68] in *Drosophila* showed that pathogenic microbes, in contrast to the normal microbiota, activate additional molecular pathways. This causes stem cells to differentiate into enteroendocrine cell types, changing the epithelial cell composition in the gut epithelium. Such changes might provide a type of memory that is based on epithelial cell structure. Accordingly, our WGCNA analysis identified differences in gene expression in the cell cycle and related processes 24 h after priming (Fig. 4A). Also, it is known from other systems that inflammatory memory exists in epithelial tissue in the form of chromatin rearrangements [6, 7]. Interesting candidates for the induction of memory and specificity of oral immune priming in our system are toxins such as Cry3Aa that are unique to certain strains of *Bt*. Previous work in *C. elegans* has indeed suggested that Cry toxins can induce specific changes in host cells via differences in the effector triggered immunity pathway [95]. The damage done by these toxins, combined with the shifts in microbiome composition, could provide the stimuli needed to invoke epigenetic or architectural changes in the gut epithelium that lead to memory in non-immune tissue.

An important evolutionary ramification of our study is that virulence factors may serve as elicitors of host immune priming. This adds host priming as a potentially important selection pressure acting on virulence factor evolution. Experimental coevolution studies have shown that Cry toxins can quickly change during coevolution of *B. thuringiensis* and *C. elegans* [96, 97] and that *Btt* evolves higher virulence variation in immune-primed *T. castaneum* hosts. Disentangling the selection pressures acting on virulence factors that both enhance pathogen infectivity and simultaneously serve as cues for host priming remains a key objective for future research.

## Conclusions

Phenomena of innate immune memory in invertebrates are collectively called immune priming. While evolutionary ancient, the specific mechanisms and properties of these phenomena appear to be different across invertebrate taxa. We show that in *T. castaneum* oral immune priming is likely triggered by damage in the gut induced by specific virulence factors (VFs) of the entomopathogen *Btt*, enabling a more targeted response to infective *Btt* spores. A role of VFs in the induction of immune priming has thus far not been described. This insight underscores the importance of using invertebrate models together with their co-evolved pathogens to explore the complex triggers and mechanisms underlying innate immune memory. Memory can arise as an emergent property of interacting cells and tissues in combination with pathogen-derived factors.

## Methods

### Model organisms

*T. castaneum* larvae used in this study stem from the Croatia 1 (Cro1) strain [30]. Cro1 was collected in 2010 in Croatia and maintained in large groups of non-overlapping generations since then. Larvae were reared on heat-sterilized (75 °C for 24 h) wheat flour (Bio Weizenmehl Type 550, dm-drogerie markt GmbH + Co. KG) including 5% Brewer’s yeast at 30 °C, 70% humidity, and a 12 h day/night rhythm.

*Bacillus thuringiensis* bv *tenebrionis* (*Btt,* BGSCID4AA1) spores were purchased from the Bacillus Genetic Stock Center of the Ohio State University (USA). *Btt Δp188* (previously denoted Btt – [34]), lacking the 188-kb plasmid, was obtained by serial passage of *Btt* at high temperatures [34].

### Study design

To understand the immediate responses following an oral priming treatment and which factors are involved in mounting a successful immune priming response, we combined proteomics data from priming media and larval size measurements, with electron microscopy and RNA-seq approaches (Fig. 1). Fourteen-day-old *T. castaneum* larvae were individually exposed to either medium- (*Bt* growth medium), priming (*Btt*)-, or control- (*Btt Δp188*) flour mixes (Priming treatment) in a well of a 96-well plate. After 24 h, larvae were transferred onto PBS flour mixes and left for another four days. Finally, larvae were transferred onto PBS- or *Btt* spore-flour mixes (Challenge treatment). For the size measurement (Fig. 1), larvae were randomly sampled before exposure to the treatment diets, and the same larvae were re-sampled before exposure to the challenge treatment. For electron microscopy and RNA-sequencing (Fig. 1), gut samples were taken from randomly sampled larvae at 3 and 24 h post-exposure to the priming treatment as well as 4 h post-exposure to the challenge treatment five days after the initial priming. In all experiments, the larvae were kept on the challenge treatment, and survival was recorded for three to five days post-exposure. Finally, the proteomes of spent culture media supernatants derived from *Btt* and *Btt Δp188* cultures were compared to narrow down the agents causing the immune priming response [34].

### Preparation of diets

The spore cultures were prepared as described in Länger and Baur et al. [34] with some modifications. In short, either 50 mL (Priming treatment) or 400 mL (Exposure treatment) *Bt* growth medium was supplemented with 250 µL or 2 mL of a salt solution and 62.5 µL or 250 µL of 1 M CaCl_2_. Lastly, 1 or 5 mL of an overnight *B. thuringiensis* culture (*Btt* or *Btt Δp188*) was added.

For producing the priming treatments, the sporulated cultures were centrifuged after 7 days twice at 2604 rcf for 15 min. The resulting supernatants were filtered through a 0.45-µm filter followed by a 0.22-µm filter to remove bacterial spores and vegetative cells. Next, the filtered supernatants were mixed with 0.15 g flour/mL of supernatant, and 10 µL of each mix was pipetted into the wells of a 96-well flat bottom plate (Greiner). Finally, the plates were put to 30 °C overnight for drying.

To produce the exposure treatment, the sporulated *Btt* cultures were centrifuged as described above. Between the first and second centrifugation the supernatants were discarded, and the spore pellets were washed in 15 mL PBS. The final spore pellets were resuspended in 5 mL PBS, the spores were counted in a Thoma counting chamber (depth 0.02 mm, square area 1/400 mm^2^) and the concentrations were adjusted to 1 × 10^10 spores/mL of PBS. Finally, 0.15 g flour was added to 1 mL of the spore/PBS mix and 10 µL of this final mix was pipetted into the wells of a 96-well flat bottom plate. The plates were put to 30 °C overnight for drying.

### Priming and challenge experiments

Priming and challenge of *T. castaneum* larvae was performed as described in [30]. In short, on day 15 after oviposition larvae were individualized into the wells of the control or priming diets (*Btt Δp188* or *Btt*) or medium control flour diets (0.15 g flour/mL *B. thuringiensis* medium). After 24 h, the larvae were transferred onto PBS diets (0.15 g flour/mL PBS) where they were left for 4 days at standard conditions (for an overview see Fig. 1). Finally, the larvae were transferred onto the exposure (*Btt* spores) or control (PBS) diets, and their survival was recorded daily for 5 days.

### Proteomics

To correlate differences in the ability to induce immune priming to differences in the proteomes of the spent growth media supernatants of *Btt* and *Btt Δp188* cultures, we used four replicate cultures of both bacterial strains (supernatants prepared as in “Preparation of priming and infection diets “). Proteins from the supernatant were directly processed for MS analysis following the SP3 protocol, using TCEP (5 mM) and CAA (14 mM) for reduction and alkylation [98]. Proteins were bound to beads by the addition of 100 µL EtOH and washed twice with 80% EtOH. They were digested in 50 mM TEAB, pH 8.5 at 37 °C. After the overnight digestion, peptides were dried by vacuum centrifugation and reconstituted in 0.5% TFA and 2% acetonitrile for LC–MS/MS analysis. An EASY-nLC 1200 (Thermo Fisher) coupled to an Exploris 480 mass spectrometer (Thermo Fisher) was used for LC–MS/MS analyses. The peptides were separated on 20-cm frit-less silica emitters (CoAnn Technologies, 0.75 µm inner diameter), packed in-house with reversed-phase ReproSil-Pur C_18_ AQ 1.9 µm resin (Dr. Maisch) and the column was constantly kept at 50 C. Mass spectra were acquired in data-dependent acquisition mode as described in Sindlinger et al. [99]. The MaxQuant software version 2.0.3.0 [100] was used to process the raw data and standard settings were used. The LFQ count was 1. The resulting MS/MS spectra were assigned to a custom *Btt* proteome assembly (*Btt* genome kindly provided by Dr. Heiko Liesegang, Institute of Microbiology and Genetics, Georg-August University of Göttingen, unpublished) with default settings and match between runs. Label-free quantification (LFQ) and intensity-based absolute quantification (iBAQ) options were enabled.

### Larval growth

To measure larval growth between priming and challenge time points, larvae were randomly selected before the transfer onto priming or control diets 14 days after oviposition and put onto petri dishes for area measurement. We measured the larval area again in the same way directly before the challenge treatment. The experiment was repeated in 3 blocks, resulting in 132 primed larvae and 135 control larvae being measured. To measure the area of the larvae, pictures were taken with an Olympus SZX12 microscope and saved as BigTIFF files. The CellSens Standard software was used to individually measure area in mm^2^. A small number of larvae that had been exposed to priming or control diets decreased in size, likely due to recent molting.

### Gut dissection

For both electron microscopy (EM) and mRNA sequencing (RNA-seq), guts of larvae were dissected under sterile conditions after the larvae were immobilized on ice. To obtain guts for EM, larvae were placed into droplets of 15 µL cold PBS and the first and last segments were removed with a scalpel. The guts were then pulled out with forceps and put into 5 mL of fixing solution (see Method section Electron microscopy). Per timepoint and treatment, five guts were dissected for EM. For the guts used for RNA-seq, 10 µL RNA-later (Thermo Fisher) was used instead of PBS. For each sample 7 guts were pooled in an 1.5-mL reaction tube containing 100 µL RNA-later on ice. Finally, the access RNA-later was removed, and the samples were shock frozen in liquid nitrogen and stored at − 80°. Per timepoint and treatment three replicates were used.

### Transmission electron microscopy

Dissected guts were fixed with 4% formaldehyde (Polysciences), 1% glutaraldehyde (Polysciences) in 0.1 M cacodylate buffer (Polysciences) pH 7.4 for 30 min and after a change for 3 h at room temperature. The samples were stored at 4 °C until further processing. After fixation, the samples were washed three times with 0.1 M cacodylate buffer (pH 7.4), post-fixed in 1% osmiumtetroxide (Polysciences) in 0.1 M cacodylate buffer at room temperature for 1 h and washed with 0.1 M cacodylate buffer. Samples were gradually dehydrated in increasing concentrations (50%, 70%, 90%, 96%, 99.8%) of ethanol (Roth) for 30 min at each step at 4 °C. After incubation with 100% propylene oxide (Serva) twice for 10 min, the samples were embedded under vacuum by subsequent incubation. Epoxy resin (Serva) was mixed with propylene oxide (1:1, 2:1 each step 4 °C overnight), epoxy resin 100% (2 × 2 h RT) and polymerized in fresh epoxy resin at 60 °C for 72 h. Ultrathin Sects. (60 nm) were generated using a Leica EM UC7 ultramicrotome. Sections were poststained with 4% uranyl acetate (Polysciences) in 25% ethanol and “Reynold’s lead citrate.” Samples were analyzed at 80 kV on a FEI-Tecnai 12 electron microscope (FEI). Images of selected areas were documented with Veleta 4 k CCD camera (emsis).

### RNA-seq

To isolate total RNA, dissected gut samples were crushed in 1 mL of TRIzol (Thermo Fisher), sonicated, and centrifuged at 13,000 RCF for 5 min at 4 °C. After a chloroform wash and another round of centrifugation at 10,500 RCF for 15 min at 4 °C, the aqueous phase was collected in EtOH and RNA lysis buffer from the SV Total RNA Isolation kit (Promega), and the manual instruction was followed. The RNA was eluted in 70 µL of RNAse-free water. Fifteen microliters of eluted RNA (~ 80 ng/µL) was sequenced by Novogene (Cambridge, UK) using the Illumina NovaSeq PE150 platform (Paired-end, 150 bp read length). Raw reads were assessed using FastQC software (version 0.11.2) [101]. Adapters were removed and reads were filtered based on criteria such as adapter contamination, nucleotide uncertainty (if > 10% of a read), or low-quality nucleotides (if Base Quality < 5 in > 50% of a read). The resulting clean reads were mapped against the *T. castaneum* genome (Tcas5.2.54, http://ftp.ensemblgenomes.org/pub/metazoa/release-54/gtf/tribolium_castaneum/) using hisat2 (version 2.0.5) [102], assembled into transcripts or genes with Stringtie (version 1.3.3b) [103] and counted with featureCounts (version 1.5.0-p3) [104].

### Statistical analysis

All statistical analyses were performed with R (version 4.3.1) [105] implemented in Rstudio (version 2023.06.1 + 524). Visualization of data was carried out using the R package “ggplot2” (version 3.4.4) [106].

For the proteome data, log_2_ transformation of LFQ intensities was performed. Only protein groups quantified in at least 3 of 4 replicates in at least one treatment supernatant (*Btt* or *Btt Δp188*) were considered for the downstream analysis and missing LFQ values were imputed based on quantile regression using the package “imputeLCMD” (version 2.1). The R package “limma” (version 3.58.1) was used to statistically test for differential expression [107]. The full proteomics table including all protein groups and their LFQ values, before and after imputation, as well as the limma statistics, can be found in Additional file 2: Table S6.

To statistically assess the influence of priming treatment on the growth of the larvae, measured area was modeled as the response variable explained by time point and treatment (priming or control) as explanatory variables and both experimental blocks and individuals as random factors using the “lmer” function from the R package “lme4” (version 1.1–35.1) [108]. The model was as follows: Size ~ Treatment*Time + (1|Block/Individual). To assess the treatment effect the full model was compared with a reduced model (Size ~ 1 + (1|Block/Individual)) using the “anova” function.

For the survival analysis of the larval growth experiment, we applied a Cox proportional hazards model with one random factor using the “coxme” function from the “coxme” package (version 2.2–18.1) [109]. The model was as follows: (Surv(Day, Event) ~ Treatment + (1|Replicate)). To assess the treatment effect, the full model was compared to a reduced model: “coxme” (Surv(Day, Event) ~ 1 + (1|Experiment), data), using the “ANOVA” function. The medium control was set as the reference. For the survival analysis of the RNA-seq and EM experiments, we applied the “coxph” function from the “survival” package (version 3.5–7) without adding a random factor: (Surv(Day, Event) ~ Treatment). In all cases, treatment (medium, *Btt* or *Btt Δp188*) was set as the fixed factor, and proportionality of the fixed effect was assessed with the cox.zph() function from the “survival” package. Additionally, the survival curves were plotted using the “survfit” function from the “survival” package [110] and “ggsurvplot” from “ggplot2.”

For the RNA-sequencing data, differential gene expression analysis was performed with DESeq2 (version 1.20.0) [35], on genes with raw counts greater than 2 in the comparison groups (priming vs. control after 3 h, 24 h, 5 days, 5 days + *Btt*). Genes with a log2 fold change > 0.5 or < − 0.5 and a False Discovery Rate (FDR, Benjamini-Hochberg) adjusted *p*-value < 0.05 were considered significantly upregulated or downregulated, respectively. GO enrichment analysis was performed using the R package clusterProfiler (version 4.10.0) [111] with a threshold of FDR adjusted *p* < 0.05. Weighted Gene Co-expression Network Analysis was carried out using the R package WGCNA (version 1.72–5) [36]. Initially, samples were checked for outliers (hierarchical clustering upon distance matrix computation and PCA), genes with a raw count lower than 10 in 50% of the samples were filtered out, and the remaining reads were variance stabilized using the “vst()” function (DESeq2). A soft thresholding power for gene correlations of 14 was chosen based on high-scale freeness and low mean connectivity. Both network type and TOMtype were set to “signed” to cluster genes that are either co-up- or co-down expressed. To statistically compare the module eigengene values of the different modules between groups, the R package limma (version 3.58.1) was used. Modules with significantly different module eigengene values (FDR adjusted *p* < 0.05) were exported to Cytoscape (version 3.10.1) [112] and visualized with the plug-in ClueGO (version 2.5.10) [113]. Only significantly enriched terms (FDR adjusted *p* < 0.05) were visualized and terms were grouped based on the number of shared genes (Kappa score = 0.4).

The R code used for the statistical analyses and for producing the figures in this manuscript can be found in Additional file 3.

## Supplementary Information

**Additional file 1. Fig. S1. Survival of T. castaneum larvae exposed to either control or priming diets from the EM and RNA-seq experiments. A** Kaplan Meier curves displaying the survival of *T. castaneum* larvae exposed to either control diets (n =96 and 121), *Bt* medium diets (n =95 and 123) or priming diets (n =96 and 123) upon exposure to infectious *Btt* spores. **B** Forest plot representing the estimated fixed-effects coefficients of primed and control compared to medium(black, dashed line at 1.0).** Fig. S2. Hierarchical cluster tree of the weighted gene co-expression analysis. **Each branch represents a single gene. Genes were clustered into modules based on their expression (unmerged), and closely related modules were merged based on their similarity in eigengene values (merged). The resulting modules are displayed in colors but were converted to numbers for later analysis.**Fig. S3. Pearson correlation of module eigengene values with control or priming treatments at the different timepoints.** Cell color = Pearson correlation coefficient. Asterisks = Significance of Student asymptotic p-value calculation (* if p < 0.05, ** if p < 0.01, *** if p < 0.001).** Fig. S4. Volcano plots for proteomic analysis. A **Volcano plot for *Btt vs.* *Btt* -cry supernatants without imputation for missing values. Plotted is the limma result for all protein groups that are at least in 3 of 4 samples of either *Btt*- or *Btt* -cry supernatants. X-axis represents the log2-fold-change (log2FC), the y-axis represents the negative log10-adjusted pvalue (-log10(adj.P.Val). The lines in the plot represent the chosen significance levels: The vertical lines are drawn at log2FC = -1/1, the horizontal line is drawn at -log10(0.05) = 1.301. Light green = significantly enriched protein groups. **. B **Volcano plot for *Btt vs. Btt* -cry supernatants with imputation for missing values. Plotted is the limma result for th imputation of all protein groups that are at least in 3 of 4 samples of either *Btt*- or *Btt* -cry supernatants. X-axis represents the log2-fold-change (log2FC), the y-axis represents the negative log10-adjusted pvalue (-log10(adj.P.Val)). The lines in the plot represent the chosen significance levels: The vertical lines are drawn at log2FC = -1/1, the horizontal line is drawn at -log10(0.05) = 1.301. Light green = significantly enriched protein groups.

**Additional file 2. Table S1. Survival and larval growth measurement data Table S2. Raw count data from RNA sequencing and raw DESeq2 results Table S3. Meta information on samples from the RNA-sequencing run Table S4. Table **including DEGs identified by DESeq2. Includes DEGs identified by DESeq2 at each timepoint between primed and control. Additionally contains categories used to group genes in biological context in the manuscript and the GO terms from the Clusterprofiler analyses.

**Additional file 3. R Markdown file for data analyses performed in this study.**

## Data Availability

Raw data for the RNA-sequencing experiment are deposited in the NCBI SRA archive under BioProject: PRJNA1174285. Raw data for the proteomics experiment can be found at the following link: https://repository.jpostdb.org/entry/JPST003396.0

## Abbreviations

*Btt Δp188*: *Bacillus thuringiensis tenebrionis* cured of the Cry toxin encoding plasmid
PBS: Phosphate buffered saline
EM: Electron microscopy
RNA-seq: RNA sequencing
DEGs: Differentially expressed genes
ME: Module Eigengene
FDR: False Discovery Rate
GO: Gene Ontology
ER: Endoplasmic reticulum
TEM: Transmission electron microscopy
PM: Peritrophic matrix
MV: Microvilli
AMPs: Antimicrobial peptides
VFs: Virulence factors
Cro1: Croatia 1 strain of *Tribolium castaneum*
LFQ: Label-Free Quantification
iBAQ: Intensity-based absolute quantification
