# Supplementary material for: Immune priming in the insect gut: a dynamic response revealed by ultrastructural and transcriptomic changes
Source: BMC Biol. 2025 Jul 28;23:227. doi: 10.1186/s12915-025-02334-4 (PMC12306008; doi:10.1186/s12915-025-02334-4)
Supplement: Supplementary file 3 — Additional file 3. R Markdown file for data analyses performed in this study. [file 12915_2025_2334_MOESM3_ESM.html]

Analyses for manuscript: Immune priming in the insect gut: A dynamic response revealed by ultrastructural and transcriptomic changes


Code 

- Show All Code
- Hide All Code

# Analyses for manuscript: Immune priming in the insect gut: A dynamic response revealed by ultrastructural and transcriptomic changes

#### Moritz Baur

#### July, 2025

- Differential
  expression analysis using DESeq2
- Creating
  tables with significantly DEGs from DESeq2
- Performing
  Weighted Gene Co-expression Network Analysis (WGCNA)
- Creating
  org.Tcastaneum.eg.db for later GO term analysis
- Manuscript
  figures
  - Fig 2: Survival and growth
    analysis
  - Fig 3: DESeq2 general
    analysis
  - Fig 4:
    WGCNA analysis
  - Fig
    5G, 6G, 7G, 8G: GO analysis for DEGs from DESeq2 at the different
    timepoints
  - Fig 9: Proteomics results
- Supplementary information
  - Additional
    file 1: Fig S1. Survival for EM and RNA-seq experiments
  - Additional file 1:
    Fig S2. WGCNA dendogram
  - Additional file
    1: FigS3. WGCNA pearson heatmap
  - Additional
    file 1: Fig S4. Proteomics volcano plot

This file includes all scripts that were used to analyze and plot the
data in the manuscript.

# Differential expression analysis using DESeq2

This was done by Novogene, but can be performed using the raw count
data sheet obtained from featureCounts and following this code:

```
library(dplyr)
library(DESeq2)
library(writexl)
library(readxl)

# prepare table to keep gene annotation for later merging
anno = read_xlsx("Additional_file_2.xlsx", sheet = 4)
anno = as.data.frame(anno)
rownames(anno) = anno$gene_id
anno = anno[, -1]
anno = anno[, 25:33]

# prepare matrix for DESeq2
countData <- read_xlsx("Additional_file_2.xlsx", sheet = 4)
countData <- countData[,1:25]
countData <- as.data.frame(countData)
rownames(countData) = countData$gene_id
countData = countData[, -1]

# P vs C after 3 hours ------------------------------------------------
countData1 = countData[, 1:6]
countData1 <- countData1  %>%
  filter(rowSums(.) >= 2)

Group1 = c("P_3h", "P_3h", "P_3h", "C_3h", "C_3h", "C_3h")
colData1 = as.data.frame(cbind(colnames(countData1), Group1))

dds1 = DESeqDataSetFromMatrix(countData = countData1, colData = colData1, design = ~Group1)
dds1 = DESeq(dds1)

# get results table and merge with annotation information
res1 = results(dds1)
res11 = as.data.frame(res1)
rows1 <- rownames(res11)
anno1 = anno[rows1,]
res12 = cbind(countData1, res11, anno1)

write_xlsx(res12, path = "PvsC_3h.xlsx")

# P vs C after 24 hours ------------------------------------------------
countData2 = countData[, 7:12]
countData2 <- countData2  %>%
  filter(rowSums(.) >= 2)

Group2 = c("P_24h", "P_24h", "P_24h", "C_24h", "C_24h", "C_24h")
colData2 = as.data.frame(cbind(colnames(countData2), Group2))

dds2 = DESeqDataSetFromMatrix(countData = countData2, colData = colData2, design = ~Group2)
dds2 = DESeq(dds2)

res2 = results(dds2)
res21 = as.data.frame(res2)

rows2 <- rownames(res21)
anno2 = anno[rows2,]
res22 = cbind(countData2, res21, anno2)

write_xlsx(res22, path = "PvsC_24h.xlsx")

# P vs C after 5 days ------------------------------------------------
countData3 = countData[, 19:24]
countData3 <- countData3  %>%
  filter(rowSums(.) >= 2)

Group3 = c("P_5d", "P_5d", "P_5d", "C_5d", "C_5d", "C_5d")
colData3 = as.data.frame(cbind(colnames(countData3), Group3))

dds3 = DESeqDataSetFromMatrix(countData = countData3, colData = colData3, design = ~Group3)
dds3 = DESeq(dds3)

res3 = results(dds3)
res31 = as.data.frame(res3)

rows3 <- rownames(res31)
anno3 = anno[rows3,]
res32 = cbind(countData3, res31, anno3)

write_xlsx(res32, path = "PvsC_5d.xlsx")

# P vs C after 5 days exposed to Btt ------------------------------------------------
countData4 = countData[, 13:18]
countData4 <- countData4  %>%
  filter(rowSums(.) >= 2)

Group4 = c("P_5d_C", "P_5d_C", "P_5d_C", "C_5d_C", "C_5d_C", "C_5d_C")
colData4 = as.data.frame(cbind(colnames(countData4), Group4))

dds4 = DESeqDataSetFromMatrix(countData = countData4, colData = colData4, design = ~Group4)
dds4 = DESeq(dds4)

res4 = results(dds4)
res41 = as.data.frame(res4)

rows4 <- rownames(res41)
anno4 = anno[rows4,]
res42 = cbind(countData4, res41, anno4)

write_xlsx(res42, path = "PvsC_5d_C.xlsx")

## All DESeq2 generated tables are included in the Additional_file_2.xlsx, sheets = 5-8 ##
```

# Creating tables with significantly DEGs from DESeq2

This will produce tables containing genes for P vs C where: pvalue
<= 0.05 and log2FC <= -0.5 or log2FC >= +0.5. An extra column
(Category) was created in which significant groups are stored that are
refered to in the manuscript.

```
library(readxl)
library(dplyr)
library(writexl)
library(stringr)

a <- read_xlsx("Additional_file_2.xlsx", sheet = 5)
b <- read_xlsx("Additional_file_2.xlsx", sheet = 6)
c <- read_xlsx("Additional_file_2.xlsx", sheet = 7)
d <- read_xlsx("Additional_file_2.xlsx", sheet = 8)

a1 = mutate(a, sig = ifelse(a$padj<0.05, "FDR < 0.05", "Not sig"))
a1[which(abs(a1$log2FoldChange)<0.5), "sig"] = "Not sig"
a1 <- subset(a1, sig =="FDR < 0.05")

b1 = mutate(b, sig = ifelse(b$padj<0.05, "FDR < 0.05", "Not sig"))
b1[which(abs(b1$log2FoldChange)<0.5), "sig"] = "Not sig"
b1 <- subset(b1, sig =="FDR < 0.05")

c1 = mutate(c, sig = ifelse(c$padj<0.05, "FDR < 0.05", "Not sig"))
c1[which(abs(c1$log2FoldChange)<0.5), "sig"] = "Not sig"
c1 <- subset(c1, sig =="FDR < 0.05")

d1 = mutate(d, sig = ifelse(d$padj<0.05, "FDR < 0.05", "Not sig"))
d1[which(abs(d1$log2FoldChange)<0.5), "sig"] = "Not sig"
d1 <- subset(d1, sig =="FDR < 0.05")

write_xlsx(a1, "DEGs_3h.xlsx")
write_xlsx(b1, "DEGs_24h.xlsx")
write_xlsx(c1, "DEGs_5d.xlsx")
write_xlsx(d1, "DEGs_5d_C.xlsx")

## All differentially expressed genes tables are included in the Additional_file_2.xlsx, sheets 10-13 ##
```

# Performing Weighted Gene Co-expression Network Analysis (WGCNA)

To perform WGCNA, genes with very low expression levels are filtered
out, samples are checked for outliers, and a soft thresholding power is
chosen before running WGCNA. Here we used the blockwiseModules function
and decided to investigate signed co-expression networks which means
that genes that tend to increase or decrease together are more likely to
merge into modlues together.

```
library(WGCNA)
library(readxl)
library(tidyverse)
library(DESeq2)
library(gridExtra)

a <- read_xlsx("Additional_file_2.xlsx", sheet = 4)
a <- a[, 1:25]
a <- a %>%
  filter(rowSums(.[, 2:25]) >= 24)
a <- column_to_rownames(a, var = "gene_id")

b <- read_xlsx("Additional_file_2.xlsx", sheet = 9)
b <- as.data.frame(b)
rownames(b) <- b$...1
b <- b[, -1]
b$Group <- as.factor(b$Group)

# Checking, filtering and normalizing samples -------------------------------------------------------------------

gsg <- goodSamplesGenes(t(a))
summary(gsg)
gsg$allOK

# plot to look for outliers
htree <- hclust(dist(t(a)), method = "average")
plot(htree)

# PCA 
pca <- prcomp(t(a))
pca.dat <- pca$x
pca.var <- pca$sdev^2

pca.var.percent <- round(pca.var/sum(pca.var)*100, digits = 2)

pca.dat <- as.data.frame(pca.dat)

ggplot(pca.dat, aes(PC1, PC2))+
  geom_point()+
  geom_text(label = row.names(pca.dat))+
  labs(x = paste0('PC1: ',pca.var.percent[1], ' %'),
       y = paste0('PC2: ',pca.var.percent[2], ' %'))+
  theme_bw()

# data normalization

colData <- b
all(row.names(colData) %in% colnames(a))
all(rownames(colData) == colnames(a))

dds <- DESeqDataSetFromMatrix(countData = a,
                              colData = colData,
                              design = ~1)

# remove low count genes (<= 10 in 50% of samples -> 0.5 x 24 = 12)
dds50 <- dds[rowSums(counts(dds) >= 10) >= 12, ] 

# normalization
dds_norm <- vst(dds50)

# vector that contains normalized and transformed count data for WGCNA
norm.counts <- assay(dds_norm) %>%
  t()

# Select soft thresholding power -------------------------------------------------------

power <- c(c(1:10), seq(from = 12, to = 50, by =2))

sft <- pickSoftThreshold(norm.counts,
                         powerVector = power,
                         networkType = "signed",
                         verbose = 5)  

sft.data <- sft$fitIndices

a1 <- ggplot(sft.data, aes(Power, SFT.R.sq, label = Power)) +
  geom_point() +
  geom_text(nudge_y = 0.1) +
  geom_hline(yintercept = 0.8, color = 'red') +
  labs(x = 'Power', y = 'Scale free topology model fit, signed R^2') +
  theme_bw()
a1

a2 <- ggplot(sft.data, aes(Power, mean.k., label = Power)) +
  geom_point() +
  geom_text(nudge_y = 0.1) +
  labs(x = 'Power', y = 'Mean Connectivity') +
  theme_classic()

grid.arrange(a1, a2, nrow = 2)
## use power of 14

# Run WGCNA ---------------------------------------------------------------

temp_cor <- cor       
cor <- WGCNA::cor

bwnet1 <- blockwiseModules(norm.counts,
                           maxBlockSize = 11000, # What size chunks (how many genes) the calculations should be run in
                           TOMType = "signed",# topological overlap matrix
                           networkType = "signed", # networkType specifies if antagonistic interactions are kept (unsigned) or not (signed)
                           power = 14, # soft threshold for network construction
                           numericLabels = TRUE,
                           mergeCutHeight = 0.25,
                           minBlockSize = 40,
                           saveTOMs = T,
                           randomSeed = 1234
)

cor <- temp_cor

# save original results
readr::write_rds(bwnet1,
                 file = file.path("WGCNA_priming.RDS"))

# Save candidate modules --------------------------------------------------

module_cand <- tibble::enframe(bwnet1$colors, name = "gene", value = "module") %>%
  # Let's add the `ME` part so its more clear what these numbers are and it matches elsewhere
  dplyr::mutate(module = paste0("ME", module))

## add gene descriptions
anno <- read_xlsx("Additional_file_2.xlsx", sheet = 4)
anno <- anno[,c(1, 33)]
module_cand <- module_cand %>%
  dplyr::inner_join(anno %>%
                      dplyr::select(gene_id, gene_description),
                    by = c("gene" = "gene_id"))

writexl::write_xlsx(module_cand, "Module_candidates.xlsx")
```

# Creating org.Tcastaneum.eg.db for later GO term analysis

We downloaded the latest annotation information for T. castaneum via
BioMart from Ensembl Metazoa to create a custom
org.Tcastaneum.eg.db.

```
library(readr)
library(dplyr)
library(readxl)
library(AnnotationDbi)
library(AnnotationForge)

# load .txt file derived from biomart ensembl
a <- read_tsv("mart_export.txt")

a %>%
  distinct(`Gene stable ID`) %>%
  summarise(unique_id_count = n())

b <- read_xlsx("Additional_file_2.xlsx", sheet = 9)

# Convert colnames of necessary columns for annotation forge 
Tc_info <- b[,c(1,1, 33 )]
Tc_info <- Tc_info[Tc_info[,2, 3]!="-",]
colnames(Tc_info) <- c("GID","SYMBOL", "GENE_INFO")

Tc_chr <- b[,c(1, 27)]
Tc_chr <- Tc_chr[Tc_chr[,2]!="-",]
colnames(Tc_chr) <- c("GID", "CHROMOSOME")

Tc_go <- a[, c(1, 8, 7)]
Tc_go <- Tc_go[Tc_go[,2]!="",]
Tc_go <- Tc_go[Tc_go[,3]!="",]
colnames(Tc_go) <- c("GID", "GO", "EVIDENCE")

Tc_go <- dplyr::distinct(Tc_go)

Tc_go <- na.omit(Tc_go)

Tc_go %>%
  distinct(`GID`) %>%
  summarise(unique_id_count = n())

# annotation forge to create org.Tcastaneum.eg.db
makeOrgPackage(gene_info=Tc_info, chromosome=Tc_chr, go=Tc_go,
               version="0.1",
               maintainer="Moritz Baur <moritz.baur6790@gmail.com>",
               author="Moritz Baur <moritz.baur6790@gmail.com>",
               outputDir = ".",
               tax_id="7070",
               genus="Tribolium",
               species="castaneum",
               goTable="go")

install.packages("./org.Tcastaneum.eg.db", repos = NULL, type = "source")
```

# Manuscript figures

## Fig 2: Survival and growth analysis

```
# Figure 2 A and B ----------------------------------------------------------------
library (survival)                                      
library(ggplot2)    
library(survminer)  
library(coxme)  
library(readxl)
library(tidyverse)
library(lmtest)

a <- read_xlsx("Additional_file_2.xlsx", sheet = 2)

a$Treatment<-as.factor(a$Treatment) 
a$Experiment<-as.factor(a$Experiment)   
a$Treatment = relevel(a$Treatment, ref = "Med-Btt") #change reference levels    

a1 <- subset(a, Experiment == 2)

# Figure 2 A --------------------------------------------------------------
S1 = survfit(Surv(Day_death, Death) ~ Treatment, data = a1)

custom_colors <- c("black", "darkolivegreen4", "darkolivegreen3")
custom_theme = theme_minimal() + theme(panel.border = element_rect(color = "black", fill = NA), axis.title.y = element_text(margin = margin(r = 10)),  # Increase distance of y-axis label from plot
                                       axis.title.x = element_text(margin = margin(t = 8)), axis.text.x = element_text(size = 9),  
                                       axis.text.y = element_text(size = 9),
                                       theme(text = element_text(family = "Arial")))

output_file <- "Fig2A.tif"
width_pixels <- 1050  
height_pixels <- 700 

tiff(filename = output_file, width = width_pixels, height = height_pixels, res = 300, units = "px")

plot <- ggsurvplot(S1, data = a1, 
                   legend.title = "Survival", 
                   xlab = "Time [days]", 
                   legend.labs = c("medium", "control", "primed"), 
                   size = 1.2, 
                   fontsize = 9,
                   palette = custom_colors, 
                   font.x = 9, 
                   font.y = 9, 
                   font.legend = 9, 
                   ggtheme = custom_theme)

print(plot)
dev.off()

# Figure 2 B ---------------------------------------------------------------

m1 = coxme(Surv(Day_death, Death) ~ Treatment + (1|Replicate), data = a1)

# ph assumption met?
cox.zph(m1)
m0 = coxme(Surv(Day_death, Death) ~ 1 + (1|Replicate), data = a1)
confint(m1) 
anova(m1,m0)    
summary(m1) 

# plot
hazard2 <- function(dat) {
  dat$Treatment = relevel(dat$Treatment, ref = "Med-Btt")
  p1 = coxme(Surv(Day_death, Death) ~ Treatment + (1|Replicate), data = dat)
  selected_treatments <- c("C-Btt", "P-Btt")
  selected_data <- dat[dat$Treatment %in% selected_treatments, ]
  coef_exp <- exp(coef(p1)) #extract the exponentiated coefficients
  conf_intervals <- confint(p1)
  conf_intervals_exp <- exp(conf_intervals)
  plot_data <- data.frame(
    Treatment = c("control", "primed"),
    exp_coef = coef_exp,
    conf_lower = conf_intervals_exp[, 1],
    conf_upper = conf_intervals_exp[, 2]
  )
  plot_data$Treatment <- factor(plot_data$Treatment, levels = c("primed", "control"))
  ggplot(plot_data, aes(x = exp_coef, y = Treatment, color = Treatment)) +
    geom_point(aes(color = Treatment), size = 3) +
    geom_errorbarh(aes(xmin = conf_lower, xmax = conf_upper, color = Treatment), height = 0.08) +
    scale_color_manual(values = c("darkolivegreen3", "darkolivegreen4"))+
    geom_vline(xintercept = 1, linetype = "dashed", color = "gray40") +
    scale_x_log10(limits = c(0.2, 3), breaks = c(0.1,0.2,0.5,1,2,3)) +  
    xlab("Estimates for hazard ratios") +
    ylab("Treatment") +
    theme_minimal() +
    theme(text = element_text(size = 9), axis.title.y = element_text(margin = margin(r = 8)),  # Increase distance of y-axis label from plot
          axis.title.x = element_text(margin = margin(t = 8)),
          theme(text = element_text(family = "Arial")),
          panel.grid.major.y = element_blank(),  
          panel.grid.minor = element_blank(),  
          panel.grid.major.x = element_line(color = "gray90"),
          panel.border = element_rect(color = "black", fill = NA),
          legend.position = "none")
}

forest_plot <- hazard2(a1)
forest_plot

# add pvalues from summary(m1)
selected_treatments <- c("C-Btt", "P-Btt")
selected_data <- a1[a1$Treatment %in% selected_treatments, ]
p_values <- c(3.4e-01, 1.1e-04)
p_values_data <- data.frame(Treatment = selected_treatments, p_value = p_values)
p_values_data$Treatment[p_values_data$Treatment == "C-Btt"] <- "control"
p_values_data$Treatment[p_values_data$Treatment == "P-Btt"] <- "primed"

plot2 <- forest_plot +
  geom_text(data = p_values_data, aes(x = 1.5, y = Treatment, label = ifelse(p_value > 0.001, paste0("p = ", round(p_value, 5)), "***")), hjust = 0, size = 3) +
  theme(plot.title = element_text(size = 9),
        theme(text = element_text(family = "Arial")))

output_file <- "Fig2B.tif"
width_pixels <- 750  
height_pixels <- 500 

tiff(filename = output_file, width = width_pixels, height = height_pixels, res = 300, units = "px")

print(plot2)
dev.off()

# Figure 2 C --------------------------------------------------------------

library(lme4)
library(lmerTest)

b <- read_xlsx("Additional_file_2.xlsx", sheet = 3)
b$Treatment <- as.factor(b$Treatment)
b$Treatment = relevel(b$Treatment, ref = "C")
b$Block <- as.factor(b$Block)
b$Individual <- as.factor(b$Individual)

b1 <- b[,c(1,3,5,7:8)]

# check for normality
b1 %>% filter(Treatment == "P") %>% pull(Size_mm2_1st) %>% shapiro.test()
b1 %>% filter(Treatment == "P") %>% pull(Size_mm2_1st) %>% hist()
b1 %>% filter(Treatment == "P") %>% pull(Size_mm2_1st) %>% mean()
b1 %>% filter(Treatment == "P") %>% pull(Size_mm2_1st) %>% sd()

b1 %>% filter(Treatment == "C") %>% pull(Size_mm2_1st) %>% shapiro.test()
b1 %>% filter(Treatment == "C") %>% pull(Size_mm2_1st) %>% hist()
b1 %>% filter(Treatment == "C") %>% pull(Size_mm2_1st) %>% mean()
b1 %>% filter(Treatment == "C") %>% pull(Size_mm2_1st) %>% sd()

b1 %>% filter(Treatment == "P") %>% pull(Size_mm2_2nd) %>% shapiro.test()
b1 %>% filter(Treatment == "P") %>% pull(Size_mm2_2nd) %>% hist()
b1 %>% filter(Treatment == "P") %>% pull(Size_mm2_2nd) %>% mean()
b1 %>% filter(Treatment == "P") %>% pull(Size_mm2_2nd) %>% sd()

b1 %>% filter(Treatment == "C") %>% pull(Size_mm2_2nd) %>% shapiro.test()
b1 %>% filter(Treatment == "C") %>% pull(Size_mm2_2nd) %>% hist()
b1 %>% filter(Treatment == "C") %>% pull(Size_mm2_2nd) %>% mean()
b1 %>% filter(Treatment == "C") %>% pull(Size_mm2_2nd) %>% sd()

# Create data table with Time as factor in long format 
b2 <- b1 %>% 
  select(Size_mm2_1st, Size_mm2_2nd, Individual) %>%
  gather(key = "Time", value = "Size", -Individual)
b3 <- b2 %>%
  inner_join(b1, by = "Individual")

# statistics
growthrate <- lmer(Size ~ Treatment*Time + (1|Block/Individual), data = b3)
growthrate2 <- lmer(Size ~ 1 + (1|Block/Individual), data = b3)

anova(growthrate, growthrate2)
summary(growthrate)

# What is the explained variance of the model
r2_nakagawa(growthrate)

# Extract the raw variances
vc <- as.data.frame(VarCorr(growthrate))$vcov

# Calculate the percentage
variance_contribution <- round((vc / sum(vc)) * 100, 2)
names(variance_contribution) <- c("Individual", "Block", "Residual")
variance_contribution

# qqplot
residuals <- resid(growthrate)
qqnorm(residuals)
qqline(residuals, col = "red")

# prepare for plot
b3$Treatment <- as.character(b3$Treatment)
b3$Treatment[b3$Treatment == "C"] <- "control"
b3$Treatment[b3$Treatment == "P"] <- "primed"
b3$Treatment <- as.factor(b3$Treatment)

# plot
plot3 <- ggplot(b3, aes(x = Time, y = Size, color = Treatment, fill = Treatment)) + 
  geom_violin(aes(group = interaction(Time, Treatment)), alpha = 0.5, position = position_dodge(width = 0.8), trim = FALSE) +
  geom_boxplot(aes(group = interaction(Time, Treatment)), width = 0.2, position = position_dodge(width = 0.8), alpha = 0.5) +
  scale_color_manual(values = c("control" = "darkolivegreen4", "primed" = "darkolivegreen3")) +
  scale_fill_manual(values = c("control" = "darkolivegreen4", "primed" = "darkolivegreen3")) +
  labs(y = expression(paste("Area [mm"^2,"]"))) +
  scale_x_discrete(labels = c("Before Treatment", "Before Challenge")) +
  theme_bw() +
  theme(text = element_text(size = 9), 
        axis.title.y = element_text(margin = margin(r = 8)),  # Increase distance of y-axis label from plot
        axis.title.x = element_text(margin = margin(t = 8)),
        theme(text = element_text(family = "Arial")))

output_file <- "Fig2C.tif"
width_pixels <- 1150 
height_pixels <- 650 

tiff(filename = output_file, width = width_pixels, height = height_pixels, res = 300, units = "px")

print(plot3)
dev.off()
```

## Fig 3: DESeq2 general analysis

This analysis consists of all DEGs that were identified with
DESeq2.

```
# Figure 3A ---------------------------------------------------------------
library(readxl)
library(dplyr)
library(ggplot2)    

a <- read_xlsx("Additional_file_2.xlsx", sheet = 10)
b <- read_xlsx("Additional_file_2.xlsx", sheet = 11)
c <- read_xlsx("Additional_file_2.xlsx", sheet = 12)
d <- read_xlsx("Additional_file_2.xlsx", sheet = 13)

a1 <-a %>%
  mutate(color_group = case_when(
    log2FoldChange > 0 ~ "Upregulated",
    log2FoldChange < 0 ~ "Downregulated",
    TRUE ~ "Other"
  ))
a1 <- a1[, c(13,24)]
a1$Time <- "3h"

b1 <-b %>%
  mutate(color_group = case_when(
    log2FoldChange > 0 ~ "Upregulated",
    log2FoldChange < 0 ~ "Downregulated",
    TRUE ~ "Other"
  ))
b1 <- b1[, c(13,24)]
b1$Time <- "24h"

c1 <-c %>%
  mutate(color_group = case_when(
    log2FoldChange > 0 ~ "Upregulated",
    log2FoldChange < 0 ~ "Downregulated",
    TRUE ~ "Other"
  ))
c1 <- c1[, c(13,24)]
c1$Time <- "5 days"

d1 <-d %>%
  mutate(color_group = case_when(
    log2FoldChange > 0 ~ "Upregulated",
    log2FoldChange < 0 ~ "Downregulated",
    TRUE ~ "Other"
  ))
d1 <- d1[, c(13,24)]
d1$Time <- "5 days +Btt"

all <- rbind(a1, b1, c1, d1)

all$Time <- factor(all$Time, levels = c("3h", "24h", "5 days","5 days +Btt"))
all$color_group <- factor(all$color_group, levels = c("Downregulated", "Upregulated"))
all1 <- all %>% group_by(color_group, Time) %>% mutate(Count = n())
all2 <- all1 %>% select(color_group, Time, Count) %>% unique()
all2$Count <- ifelse(all2$color_group == "Downregulated", -all2$Count, all2$Count)

plot1 <- ggplot(all2, aes(x = Time, y = Count, fill = color_group)) +
  geom_bar(stat = "identity", position = "dodge", colour = "black", size = 1) +
  labs(x = "", y = "Number of DEGs", title = "", fill = "Regulation:") +
  scale_fill_manual(values = c("Upregulated" = "#e41a1c", "Downregulated" = "#377eb8"), labels = c("Down", "Up")) +
  scale_x_discrete(labels = c("3 hours", "24 hours", "5 days", "5 days +Btt")) +
  scale_y_continuous(limits = c(-600, 600),
                     breaks = seq(-600, 600, by = 200),
                     labels = abs(seq(-600, 600, by = 200))) +
  theme_bw() +
  theme(text = element_text(size = 10),
        legend.position = "bottom",  
        legend.direction = "horizontal",
        legend.margin = margin(t = -15),
        theme(text = element_text(family = "Arial")))
 
output_file <- "Fig3A.tif"
width_pixels <- 750  
height_pixels <- 600 

tiff(filename = output_file, width = width_pixels, height = height_pixels, res = 300, units = "px")

print(plot1)
dev.off()

# Figure 3B + 3C ----------------------------------------------------------

 library(ggvenn)
 library(scales)
 library(readxl)
 library(dplyr)
 
 ## Load genes that are DEGs in modules
e <- read_xlsx("Additional_file_2.xlsx", sheet = 10)
f <- read_xlsx("Additional_file_2.xlsx", sheet = 11)
g <- read_xlsx("Additional_file_2.xlsx", sheet = 12)
h <- read_xlsx("Additional_file_2.xlsx", sheet = 13)
 
 le2 <- e %>% select(gene_id, log2FoldChange) %>% mutate(regulation = ifelse(e$log2FoldChange > 0, "Up", "Down"))
 le3 <- le2 %>% filter(regulation == "Up") %>% select(gene_id)
 le3 <- le3$gene_id
 le4 <- le2 %>% filter(regulation == "Down") %>% select(gene_id)
 le4 <- le4$gene_id
 
 lf2 <- f %>% select(gene_id, log2FoldChange) %>% mutate(regulation = ifelse(f$log2FoldChange > 0, "Up", "Down"))
 lf3 <- lf2 %>% filter(regulation == "Up") %>% select(gene_id)
 lf3 <- lf3$gene_id
 lf4 <- lf2 %>% filter(regulation == "Down") %>% select(gene_id)
 lf4 <- lf4$gene_id
 
 lg2 <- g %>% select(gene_id, log2FoldChange) %>% mutate(regulation = ifelse(g$log2FoldChange > 0, "Up", "Down"))
 lg3 <- lg2 %>% filter(regulation == "Up") %>% select(gene_id)
 lg3 <- lg3$gene_id
 lg4 <- lg2 %>% filter(regulation == "Down") %>% select(gene_id)
 lg4 <- lg4$gene_id
 
 lh2 <- h %>% select(gene_id, log2FoldChange) %>% mutate(regulation = ifelse(h$log2FoldChange > 0, "Up", "Down"))
 lh3 <- lh2 %>% filter(regulation == "Up") %>% select(gene_id)
 lh3 <- lh3$gene_id
 lh4 <- lh2 %>% filter(regulation == "Down") %>% select(gene_id)
 lh4 <- lh4$gene_id
 
 ## Fig 3B
 venn_plot_up <- ggvenn(
   list(
     "3 hours" = le3,
     "24 hours" = lf3,
     "5 days" = lg3,
     "5 days exp." = lh3
   ), show_percentage = F, set_name_size = 0, stroke_size = 0.5, text_size = 3
 )
 
 plot3 <- venn_plot_up +
   scale_fill_manual(values = c("#e41a1c80", "#e41a1c80","#e41a1c80", "#e41a1c80"))+
   labs(title = "")+
   theme(
     text = element_text(size = 8),
     theme(text = element_text(family = "Arial")))+
   annotate("text", x = -1, y = 1, label = "24 hours", size = 3, vjust = -1) +
   annotate("text", x = 1, y = 1, label = "5 days", size = 3, vjust = -1) +
   annotate("text", x = -1.5, y = -1, label = "3 hours", size = 3, vjust = 3) +
   annotate("text", x = 1.5, y = -1, label = "5 days +Btt", size = 3, vjust = 3)
 
 output_file <- "Fig3B.tif"
 width_pixels <- 700  
 height_pixels <- 600 
 
 tiff(filename = output_file, width = width_pixels, height = height_pixels, res = 300, units = "px")
 
 print(plot3)
 dev.off()
 
 ## Fig 3C
 venn_plot_down <- ggvenn(
   list(
     "3 hours" = le4,
     "24 hours" = lf4,
     "5 days" = lg4,
     "5 days exp." = lh4
   ), show_percentage = F, set_name_size = 0, stroke_size = 0.5, text_size = 3
 )
 
plot4 <- venn_plot_down +
   scale_fill_manual(values = c("#377eb880","#377eb880","#377eb880","#377eb880"))+
   labs(title = "")+
   theme(
     text = element_text(size = 8),
     theme(text = element_text(family = "Arial")))+
   annotate("text", x = -1, y = 1, label = "24 hours", size = 3, vjust = -1) +
   annotate("text", x = 1, y = 1, label = "5 days", size = 3, vjust = -1) +
   annotate("text", x = -1.5, y = -1, label = "3 hours", size = 3, vjust = 3) +
   annotate("text", x = 1.5, y = -1, label = "5 days +Btt", size = 3, vjust = 3)
 
 output_file <- "Fig3C.tif"
 width_pixels <- 700  
 height_pixels <- 600 
 
 tiff(filename = output_file, width = width_pixels, height = height_pixels, res = 300, units = "px")
 
 print(plot4)
 dev.off()
 
 # Figure 3D ---------------------------------------------------------------
 
 library(DESeq2)
 library(ComplexHeatmap)
 library(colorspace)
 library(stringr)
 
 a <- read_xlsx("Additional_file_2.xlsx", sheet = 4)
 
 # filter data for genes used in the DESeq2 analyses
 countData <- read_xlsx("Additional_file_2.xlsx", sheet = 4)
 
 deseq3h <- read_xlsx("Additional_file_3.xlsx", sheet = 5)
 deseq24h <- read_xlsx("Additional_file_3.xlsx", sheet = 6)
 deseq5d <- read_xlsx("Additional_file_3.xlsx", sheet = 7)
 deseq5d_e <- read_xlsx("Additional_file_3.xlsx", sheet = 8)
 
 ids3h <- deseq3h$gene_name
 ids24h <- deseq24h$gene_name
 ids5d <- deseq5d$gene_name
 ids5de <- deseq5d_e$gene_name
 
 countData <- subset(countData, gene_id %in% ids3h | gene_id %in% ids24h | gene_id %in% ids5d | gene_id %in% ids5de)
 
 # normalization using DESeq2 vst()
 countData <- countData[,1:25]
 countData <- as.data.frame(countData)
 rownames(countData) = countData$gene_id
 countData = countData[, -1]
 
 Group = c("P_3h", "P_3h", "P_3h", "C_3h", "C_3h", "C_3h",
           "P_24h", "P_24h", "P_24h", "C_24h", "C_24h", "C_24h",
           "P_5d_C", "P_5d_C", "P_5d_C", "C_5d_C", "C_5d_C", "C_5d_C",
           "P_5d", "P_5d", "P_5d", "C_5d", "C_5d", "C_5d")
 
 colData = as.data.frame(cbind(colnames(countData), Group))
 dds = DESeqDataSetFromMatrix(countData = countData, colData = colData, design = ~1) 
 
 dds_norm <- vst(dds)
 
 norm.counts <- as.data.frame(assay(dds_norm))
 norm.counts$gene_id <- rownames(norm.counts)
 
 # filter normalized data for significant genes
b <- read_xlsx("Additional_file_2.xlsx", sheet = 10)
c <- read_xlsx("Additional_file_2.xlsx", sheet = 11)
d <- read_xlsx("Additional_file_2.xlsx", sheet = 12)
e <- read_xlsx("Additional_file_2.xlsx", sheet = 13)
 
 lb <- b$gene_id
 lc <- c$gene_id
 ld <- d$gene_id
 le <- e$gene_id
 
 lall <- subset(norm.counts, gene_id %in% lb | gene_id %in% lc | gene_id %in% ld | gene_id %in% le)
 all <- lall %>%
   distinct(`gene_id`, .keep_all = TRUE)
 
 # Prepare matrix
 analysis <- all
 analysis <- analysis[, 1:25]
 analysis <- as.data.frame(analysis)
 rownames(analysis) <- analysis$gene_id
 analysis = analysis[, -25]
 analysis <- as.matrix(analysis)
 analysis <- analysis[complete.cases(analysis), ]
 analysis <- analysis %>%
   t() %>%
   scale() %>%
   t()
 
 # add the column annotations in the heatmap
 ann <- data.frame(col = colnames(analysis))
 ann$Treatment <- ifelse(substr(ann$col, 1, 1) == "P", "primed",
                         ifelse(substr(ann$col, 1, 1) == "C", "control", "none"))
 ann <- ann %>%
   mutate(Time = case_when(
     grepl("_(\\d+)h_(\\d+)", col, perl = TRUE) ~ str_c(str_extract(col, "\\d+"), " hours"),
     grepl("_(\\d+)d_(\\d+)", col, perl = TRUE) ~ str_c(str_extract(col, "\\d+"), " days"),
     grepl("_(\\d+)d_[a-zA-Z]_(\\d+)", col, perl = TRUE) ~ str_c(str_extract(col, "\\d+"), " days"),
     TRUE ~ col  # if none of the patterns match, keep the original value
   ))
 ann <- ann %>%
   mutate(Btt_Exposure = case_when(
     grepl("_(\\d+)d_C", col, perl = TRUE) ~ "Yes",
     TRUE ~ "No"  # if none of the patterns match, keep the original value
   )) 
 
 colors <- list('Treatment' = c('primed' = 'darkolivegreen3', 'control' = 'darkolivegreen4'),
                'Time' = c( '3 hours' = 'grey88', '24 hours' = 'grey', '5 days' = 'grey46'),
                'Btt_Exposure' = c('Yes' = 'gold1', 'No' = 'papayawhip'))
 
 ann2 <- ann[,2:4]
 ann2$Time <- factor(ann2$Time, levels = c("3 hours", "24 hours", "5 days"))
 ann2$Treatment <- factor(ann2$Treatment, levels = c("control", "primed"))
 
 colAnn <- HeatmapAnnotation(df = ann2,
                             which = 'col',
                             col = colors,
                             annotation_width = unit(c(0.4, 2), 'cm'),
                             gap = unit(1, 'mm'),
                             annotation_name_gp= gpar(fontsize = 10),
                             annotation_legend_param = list(
                               title_gp = gpar(fontsize = 9),
                               labels_gp = gpar(fontsize = 9)
                             ))
 
 min_value <- round(min(analysis), 1)
 max_value <- round(max(analysis), 1)
 middle_value <- round((min_value + max_value) / 2, 1)
 
 color_func <- circlize::colorRamp2(
   c(min_value,middle_value , max_value),
   c("#377eb8", "white", "#e41a1c"))
 
 colnames(analysis) <- substr(colnames(analysis), 1, 1)
 
 # plot
 heatmap <- ComplexHeatmap::Heatmap(
   analysis,
   col = color_func,
   cluster_columns = F,
   show_row_names = F,
   row_names_side = "left",
   show_column_names = F,
   use_raster =F,  
   cluster_rows = T,  
   heatmap_legend_param = list(
     at = c(min_value, middle_value, max_value),
     legend_height = unit(3, "cm"),
     grid_width = unit(0.2, "cm"),
     title = "",
     labels_gp = gpar(fontsize = 9)),
   top_annotation=colAnn,
   column_title ="Clustering based on DEGs",
   column_names_rot = 0,
   column_names_centered = T
 )
 
 plot2 <- draw(heatmap, heatmap_legend_side="right",
      align_heatmap_legend = "heatmap_center")
 
 output_file <- "Fig3D.tif"
 width_pixels <- 1400  
 height_pixels <- 1500 
 
 tiff(filename = output_file, width = width_pixels, height = height_pixels, res = 300, units = "px")
 
 print(plot2)
 dev.off()
 
 # Figure 3E ----------------------------------------------------------
 
library(ggplot2)
library(ggrepel)
library(plotly)
library(tidyverse)
library(ggalt)
library(readxl)
library(DESeq2)
library(stringr)
library(viridisLite)
library(processx)


a <- read_xlsx("Additional_file_2.xlsx", sheet =4)

# filter data for genes used in the DESeq2 analyses
countData <- read_xlsx("Additional_file_2.xlsx", sheet = 4)

deseq3h <- read_xlsx("Additional_file_2.xlsx", sheet = 5)
deseq24h <- read_xlsx("Additional_file_2.xlsx", sheet = 6)
deseq5d <- read_xlsx("Additional_file_2.xlsx", sheet = 7)
deseq5d_e <- read_xlsx("Additional_file_2.xlsx", sheet = 8)

ids3h <- deseq3h$gene_name
ids24h <- deseq24h$gene_name
ids5d <- deseq5d$gene_name
ids5de <- deseq5d_e$gene_name

countData <- subset(countData, gene_id %in% ids3h | gene_id %in% ids24h | gene_id %in% ids5d | gene_id %in% ids5de)

# normalization using DESeq2 vst()
countData <- countData[,1:25]
countData <- as.data.frame(countData)
rownames(countData) = countData$gene_id
countData = countData[, -1]

Group = c("P_3h", "P_3h", "P_3h", "C_3h", "C_3h", "C_3h",
          "P_24h", "P_24h", "P_24h", "C_24h", "C_24h", "C_24h",
          "P_5d_C", "P_5d_C", "P_5d_C", "C_5d_C", "C_5d_C", "C_5d_C",
          "P_5d", "P_5d", "P_5d", "C_5d", "C_5d", "C_5d")

colData = as.data.frame(cbind(colnames(countData), Group))
dds = DESeqDataSetFromMatrix(countData = countData, colData = colData, design = ~1) 

dds_norm <- vst(dds)

norm.counts <- as.data.frame(assay(dds_norm))
norm.counts$gene_id <- rownames(norm.counts)

# filter normalized data for significant genes
b <- read_xlsx("Additional_file_2.xlsx", sheet = 5)
c <- read_xlsx("Additional_file_2.xlsx", sheet = 6)
d <- read_xlsx("Additional_file_2.xlsx", sheet = 7)
e <- read_xlsx("Additional_file_2.xlsx", sheet = 8)

lb <- b$gene_id
lc <- c$gene_id
ld <- d$gene_id
le <- e$gene_id

lall <- subset(norm.counts, gene_id %in% lb | gene_id %in% lc | gene_id %in% ld | gene_id %in% le)
all <- lall %>%
  distinct(`gene_id`, .keep_all = TRUE)

all_long <- gather(all, key = "Sample", value = "Expression", -gene_id)

all_long <- all_long %>%
  mutate(Group = sub("_[^_]+$", "", Sample))

all_pca <- all[,1:24]
all_pca <- all_pca[complete.cases(all_pca), ]
all_pca2 <- t(all_pca)

b <- read_xlsx("Additional_file_2.xlsx", sheet = 9)
b <- as.data.frame(b)
rownames(b) <- b$Sample

pca_result <- prcomp(all_pca2[, 1:ncol(all_pca2)], scale = T)
pcs <- pca_result$x
pca_df <- data.frame(PC1 = pcs[, 1], PC2 = pcs[, 2], PC3 = pcs[, 3], SampleID = rownames(b))
pca_df$Group <- b$Group
pca_df$Treatment <- b$Treatment
pca_df$Exposure <- b$Exposure
pca_df <- pca_df %>%
  mutate(GroupCategory = case_when(
    grepl("^P|^C", Group) & Exposure == "Yes" ~ "Btt",  # Category "Btt" for Group starting with "P" or "C" and Exposure is "Yes"
    grepl("^P_", Group) ~ "P",                         # Category "P" for entries starting with "P_"
    grepl("^C_", Group) ~ "C"                          # Category "C" for entries starting with "C_"
  ))
explained_variance <- pca_result$sdev^2 / sum(pca_result$sdev^2) * 100

pca_df$Group <- factor(pca_df$Group, levels = c("P_3h", "C_3h", "P_24h", "C_24h",
                                                "P_5d_C", "C_5d_C", "P_5d", "C_5d"))

pca_df$Time <- sub("^[A-Z]+_", "", pca_df$Group)
pca_df$Time <- factor(pca_df$Time, levels = c("3h", "24h","5d", "5d_C"))

write_xlsx(pca_df, "pca_python.xlsx")

#### === Python === ###

import pandas as pd
import numpy as np
from sklearn.decomposition import PCA
import plotly.graph_objects as go
import plotly.express as px

pca_df = pd.read_excel("pca_python.xlsx")

# === Plot with Plotly ===

custom_colors = {
    "P_3h": "#a2cd5a", 
    "C_3h": "#6e8b3d",
    "P_24h": "#a2cd5a",      # You can define colors for all group categories
    "C_24h": "#6e8b3d",
    "P_5d_C": "#a2cd5a",
    "C_5d_C": "#6e8b3d",
    "P_5d": "#a2cd5a",
    "C_5d": "#6e8b3d"
}

custom_shapes = {"3h": "circle", "24h": "square", "5d": "diamond", "5d_C": "cross"}

fig = px.scatter_3d(
    pca_df, 
    x='PC1', 
    y='PC2', 
    z='PC3',
    color='Group',
    symbol='Time',
    color_discrete_map=custom_colors,
    symbol_map=custom_shapes
)

# === Customize Layout ===
fig.update_traces(
    marker=dict(
        size=5,  # Change marker size here
        line=dict(width=1, color='black')  # Add black border to points
    )
)

fig.update_layout(
    # Rename axes to show % variance
    scene=dict(
        xaxis_title=f"PC1 ({round(pca_df['Variance_exp'][0],2)}%)",
        yaxis_title=f"PC2 ({round(pca_df['Variance_exp'][1],2)}%)",
        zaxis_title=f"PC3 ({round(pca_df['Variance_exp'][2],2)}%)",
        xaxis=dict(backgroundcolor='white', gridcolor='lightgrey', showbackground=True),
        yaxis=dict(backgroundcolor='white', gridcolor='lightgrey', showbackground=True),
        zaxis=dict(backgroundcolor='white', gridcolor='lightgrey', showbackground=True),
    ),
    
    # White background for the whole plot
    paper_bgcolor='white',
    plot_bgcolor='white',
    
    # Font size and style
    font=dict(
        family="Arial, sans-serif",
        size=14,  # base font size
        color="black"
    )
)
fig.update_layout(
    showlegend=False,  # Hide legend
    width=800,  # Width of the figure
    height=600   # Height of the figure
)

def set_camera(angle_deg=45, distance=2, height=1.2):
    angle = np.deg2rad(angle_deg)
    return dict(
        eye=dict(
            x=distance * np.cos(angle),
            y=distance * np.sin(angle),
            z=height
        ),
        up=dict(x=0, y=0, z=1),
        center=dict(x=0, y=0, z=0)
    )

fig.update_layout(
    scene_camera=set_camera(angle_deg=50, distance=0.8, height=2)
)

fig.show()

# === Save snapshot as static image ===
fig.write_image("Fig3E.png", scale=4)
```

## Fig 4: WGCNA analysis

Significant modules were identified by statistically comparing the
module eigengene values between treatments using limma. The module
eigengene values of significant modules were plotted for comparison. The
gene ids within significant modules were exported to Cytoscape and GO
analysis was performed for each module using the plug-in ClueGO.

```
library(WGCNA)
library(tidyverse)
library(readxl)
library(limma)
library(writexl)

a <- read_xlsx("Additional_file_2.xlsx", sheet = 4)
a <- a[, 1:25]
a <- a %>%
  filter(rowSums(.[, 2:25]) >= 24)
a <- column_to_rownames(a, var = "gene_id")

b <- read_xlsx("Additional_file_2.xlsx", sheet = 9)
b <- as.data.frame(b)
rownames(b) <- b$...1
b <- b[, -1]
b$Group <- as.factor(b$Group)

# Analysis: Pairwise Limma -----------------------------------------

## open file
bwnet1 <- readr::read_rds("WGCNA_priming.RDS")

## inspect
module_eigengenes <- bwnet1$MEs
head(module_eigengenes)

## pairwise limma

module_eigengenes <- module_eigengenes[, 1:25]

# PvsC_3h
b1 <- b[1:6,]
desired_levels <- c("P_3h", "C_3h")
b1$Group <- factor(b1$Group, levels = desired_levels)

module1 <- module_eigengenes[1:6, ]

des_mat1 <- model.matrix(~b1$Group)

fit <- limma::lmFit(t(module1), design = des_mat1)
fit <- limma::eBayes(fit)

stats_df <- limma::topTable(fit, number = ncol(module1)) %>%
  tibble::rownames_to_column("module")
head(stats_df)

write_xlsx(stats_df, "stats_modules_3h.xlsx")

# PvsC_24h
b2 <- b[7:12,]
desired_levels <- c("P_24h", "C_24h")
b2$Group <- factor(b2$Group, levels = desired_levels)

module2 <- module_eigengenes[7:12,]

des_mat2 <- model.matrix(~b2$Group)

fit <- limma::lmFit(t(module2), design = des_mat2)
fit <- limma::eBayes(fit)

stats_df <- limma::topTable(fit, number = ncol(module2)) %>%
  tibble::rownames_to_column("module")
head(stats_df)

write_xlsx(stats_df, "stats_modules_24h.xlsx")

# PvsC_5d Btt exposed
b3 <- b[13:18,]
desired_levels <- c("P_5d_C", "C_5d_C")
b3$Group <- factor(b3$Group, levels = desired_levels)

module3 <- module_eigengenes[13:18,]

des_mat3 <- model.matrix(~b3$Group)

fit <- limma::lmFit(t(module3), design = des_mat3)
fit <- limma::eBayes(fit)

stats_df <- limma::topTable(fit, number = ncol(module3)) %>%
  tibble::rownames_to_column("module")
head(stats_df)

write_xlsx(stats_df, "stats_modules_5d_C.xlsx")

# PvsC_5d
b4 <- b[19:24,]
desired_levels <- c("P_5d", "C_5d")
b4$Group <- factor(b4$Group, levels = desired_levels)

module4 <- module_eigengenes[19:24,]

des_mat4 <- model.matrix(~b4$Group)

fit <- limma::lmFit(t(module4), design = des_mat4)
fit <- limma::eBayes(fit)

stats_df <- limma::topTable(fit, number = ncol(module4)) %>%
  tibble::rownames_to_column("module")
head(stats_df)

write_xlsx(stats_df, "stats_modules_5d.xlsx")

# Plotting significant modules --------------------------------------------

##candidates: M1, M2, M11, M13, M14, M17
allmodules <- module_eigengenes %>%
  tibble::rownames_to_column("Samples")

candmod <- allmodules

candmod <- candmod %>%
  dplyr::inner_join(b %>%
                      dplyr::select(Sample, Group),
                    by = c("Samples" = "Sample"))

neworder1 <- c("P_3h", "C_3h", "P_24h", "C_24h", "P_5d", "C_5d","P_5d_C", "C_5d_C")
candmod$Group <- factor(candmod$Group, levels = neworder1)
candmod <- candmod %>%
  mutate(Time = as.factor(ifelse(grepl("_C", Group), sub(".*_(\\d+)(h)?.*", "\\1 d +Btt", Group), 
                                 ifelse(grepl("(\\d+)d", Group), sub(".*_(\\d+)(h)?.*", "\\1 d", Group),sub(".*_(\\d+)(h)?.*", "\\1 h", Group)))))
candmod <- candmod %>%
  mutate(Treatment = ifelse(grepl("^P", Group), "primed", "control"))
candmod$Time <- factor(candmod$Time, levels = c("3 h", "24 h", "5 d", "5 d +Btt"))
candmod$Treatment <- factor(candmod$Treatment, levels = c("control", "primed"))

custom_colors <- c("primed" = "darkolivegreen3", "control" = "darkolivegreen4")

# Module 1
plot1 <- ggplot(candmod, aes(Treatment, ME1, color = Treatment)) +
  geom_jitter(size = 2, width = 0.1)+
  facet_wrap(~ Time, ncol = 4, nrow = 1)+
  scale_color_manual(values = custom_colors)+
  geom_hline(
    aes(yintercept = mean(ME1)),
    linetype = "dashed",
    color = "black",
    size = 0.5)+
  scale_y_continuous(
    limits = c(-0.5, 0.5),
    breaks = c(-0.5, -0.25, 0, 0.25, 0.5),
    labels = c("-0.5", "-0.25", "0", "0.25", "0.5")
  ) +
  geom_segment(
    data = subset(candmod, Time == "24 h"), 
    aes(x = 1, xend = 2, y = 0.25, yend = 0.25), 
    color = "black", 
    size = 0.5
  ) +
  geom_text(
    data = subset(candmod, Time == "24 h"), 
    aes(x = 1.5, y = 0.3, label = "*"), 
    size = 3, 
    color = "black"
  ) +
  theme_bw()+
  theme(
    text = element_text(size = 8),
    legend.position = "bottom",  
    legend.direction = "horizontal",
    legend.margin = margin(t = -20),
    axis.text.x = element_blank())+  # Remove the legend
  labs(x="", y ="ME")
plot1

output_file <- "Fig4A_1.tif"
width_pixels <- 720  
height_pixels <- 500 

tiff(filename = output_file, width = width_pixels, height = height_pixels, res = 300, units = "px")

print(plot1)
dev.off()

# Module 2
plot2 <- ggplot(candmod, aes(Treatment, ME2, color = Treatment)) +
  geom_jitter(size = 2, width = 0.1)+
  facet_wrap(~ Time, ncol = 4, nrow = 1)+
  scale_color_manual(values = custom_colors)+
  geom_hline(
    aes(yintercept = mean(ME2)),
    linetype = "dashed",
    color = "black",
    size = 0.5)+
  scale_y_continuous(
    limits = c(-0.5, 0.5),
    breaks = c(-0.5, -0.25, 0, 0.25, 0.5),
    labels = c("-0.5", "-0.25", "0", "0.25", "0.5")
  ) +
  geom_segment(
    data = subset(candmod, Time == "24 h"), 
    aes(x = 1, xend = 2, y = 0.375, yend = 0.375), 
    color = "black", 
    size = 0.5
  ) +
  geom_text(
    data = subset(candmod, Time == "24 h"), 
    aes(x = 1.5, y = 0.425, label = "*"), 
    size = 3, 
    color = "black"
  ) +
  theme_bw()+
  theme(
    text = element_text(size = 8),
    legend.position = "bottom",  
    legend.direction = "horizontal",
    legend.margin = margin(t = -20),
    axis.text.x = element_blank())+  # Remove the legend
  labs(x="", y ="ME")
plot2

output_file <- "Fig4B_1.tif"
width_pixels <- 720  
height_pixels <- 500 

tiff(filename = output_file, width = width_pixels, height = height_pixels, res = 300, units = "px")

print(plot2)
dev.off()

# Module 11
plot3 <- ggplot(candmod, aes(Treatment, ME11, color = Treatment)) +
  geom_jitter(size = 2, width = 0.1)+
  facet_wrap(~ Time, ncol = 4, nrow = 1)+
  scale_color_manual(values = custom_colors)+
  geom_hline(
    aes(yintercept = mean(ME11)),
    linetype = "dashed",
    color = "black",
    size = 0.5)+
  scale_y_continuous(
    limits = c(-0.5, 0.5),
    breaks = c(-0.5, -0.25, 0, 0.25, 0.5),
    labels = c("-0.5", "-0.25", "0", "0.25", "0.5")
  ) +
  geom_segment(
    data = subset(candmod, Time == "24 h"), 
    aes(x = 1, xend = 2, y = 0.45, yend = 0.45), 
    color = "black", 
    size = 0.5
  ) +
  geom_text(
    data = subset(candmod, Time == "24 h"), 
    aes(x = 1.5, y = 0.5, label = "*"), 
    size = 3, 
    color = "black"
  ) +
  theme_bw()+
  theme(
    text = element_text(size = 8),
    legend.position = "none",  
    legend.direction = "horizontal",
    legend.margin = margin(t = -20),
    axis.text.x = element_blank())+  # Remove the legend
  labs(x="", y ="ME")
plot3

output_file <- "Fig4B_2.tif"
width_pixels <- 720  
height_pixels <- 450 

tiff(filename = output_file, width = width_pixels, height = height_pixels, res = 300, units = "px")

print(plot3)
dev.off()

# Module 13
plot4 <- ggplot(candmod, aes(Treatment, ME13, color = Treatment)) +
  geom_jitter(size = 2, width = 0.1)+
  facet_wrap(~ Time, ncol = 4, nrow = 1)+
  scale_color_manual(values = custom_colors)+
  geom_hline(
    aes(yintercept = mean(ME13)),
    linetype = "dashed",
    color = "black",
    size = 0.5)+
  scale_y_continuous(
    limits = c(-0.5, 0.5),
    breaks = c(-0.5, -0.25, 0, 0.25, 0.5),
    labels = c("-0.5", "-0.25", "0", "0.25", "0.5")
  ) +
  geom_segment(
    data = subset(candmod, Time == "24 h"), 
    aes(x = 1, xend = 2, y = 0.125, yend = 0.125), 
    color = "black", 
    size = 0.5
  ) +
  geom_text(
    data = subset(candmod, Time == "24 h"), 
    aes(x = 1.5, y = 0.175, label = "*"), 
    size = 3, 
    color = "black"
  ) +
  theme_bw()+
  theme(
    text = element_text(size = 8),
    legend.position = "bottom",  
    legend.direction = "horizontal",
    legend.margin = margin(t = -20),
    axis.text.x = element_blank())+  # Remove the legend
  labs(x="", y ="ME")
plot4

output_file <- "Fig4A_2.tif"
width_pixels <- 720  
height_pixels <- 500 

tiff(filename = output_file, width = width_pixels, height = height_pixels, res = 300, units = "px")

print(plot4)
dev.off()

# Module 14
plot5 <- ggplot(candmod, aes(Treatment, ME14, color = Treatment)) +
  geom_jitter(size = 2, width = 0.1)+
  facet_wrap(~ Time, ncol = 4, nrow = 1)+
  scale_color_manual(values = custom_colors)+
  geom_hline(
    aes(yintercept = mean(ME14)),
    linetype = "dashed",
    color = "black",
    size = 0.5)+
  scale_y_continuous(
    limits = c(-0.5, 0.5),
    breaks = c(-0.5, -0.25, 0, 0.25, 0.5),
    labels = c("-0.5", "-0.25", "0", "0.25", "0.5")
  ) +
  geom_segment(
    data = subset(candmod, Time == "24 h"), 
    aes(x = 1, xend = 2, y = 0.4, yend = 0.4), 
    color = "black", 
    size = 0.5
  ) +
  geom_text(
    data = subset(candmod, Time == "24 h"), 
    aes(x = 1.5, y = 0.45, label = "**"), 
    size = 3, 
    color = "black"
  ) +
  geom_segment(
    data = subset(candmod, Time == "5 d"), 
    aes(x = 1, xend = 2, y = 0.4, yend = 0.4), 
    color = "black", 
    size = 0.5
  ) +
  geom_text(
    data = subset(candmod, Time == "5 d"), 
    aes(x = 1.5, y = 0.45, label = "*"), 
    size = 3, 
    color = "black"
  ) +
  theme_bw()+
  theme(
    text = element_text(size = 8),
    legend.position = "bottom",  
    legend.direction = "horizontal",
    legend.margin = margin(t = -20),
    axis.text.x = element_blank())+  # Remove the legend
  labs(x="", y ="")
plot5

output_file <- "Fig4B_3.tif"
width_pixels <- 720  
height_pixels <- 500 

tiff(filename = output_file, width = width_pixels, height = height_pixels, res = 300, units = "px")

print(plot5)
dev.off()

# Module 17
plot6 <- ggplot(candmod, aes(Treatment, ME17, color = Treatment)) +
  geom_jitter(size = 2, width = 0.1)+
  facet_wrap(~ Time, ncol = 4, nrow = 1)+
  scale_color_manual(values = custom_colors)+
  geom_hline(
    aes(yintercept = mean(ME17)),
    linetype = "dashed",
    color = "black",
    size = 0.5)+
  scale_y_continuous(
    limits = c(-0.5, 0.5),
    breaks = c(-0.5, -0.25, 0, 0.25, 0.5),
    labels = c("-0.5", "-0.25", "0", "0.25", "0.5")
  ) +
  geom_segment(
    data = subset(candmod, Time == "24 h"), 
    aes(x = 1, xend = 2, y = 0.45, yend = 0.45), 
    color = "black", 
    size = 0.5
  ) +
  geom_text(
    data = subset(candmod, Time == "24 h"), 
    aes(x = 1.5, y = 0.5, label = "*"), 
    size = 3, 
    color = "black"
  ) +
  geom_segment(
    data = subset(candmod, Time == "5 d"), 
    aes(x = 1, xend = 2, y = 0.45, yend = 0.45), 
    color = "black", 
    size = 0.5
  ) +
  geom_text(
    data = subset(candmod, Time == "5 d"), 
    aes(x = 1.5, y = 0.5, label = "*"), 
    size = 3, 
    color = "black"
  ) +
  theme_bw()+
  theme(
    text = element_text(size = 8),
    legend.position = "none",  
    legend.direction = "horizontal",
    legend.margin = margin(t = -20),
    axis.text.x = element_blank())+  # Remove the legend
  labs(x="", y ="")
plot6

output_file <- "Fig4B_4.tif"
width_pixels <- 720  
height_pixels <- 450 

tiff(filename = output_file, width = width_pixels, height = height_pixels, res = 300, units = "px")

print(plot6)
dev.off()
```

## Fig 5G, 6G, 7G, 8G: GO analysis for DEGs from DESeq2 at the different timepoints

The clusterprofiler command was run subseqeuently for the different
DEGs files (representing the different timepoints).

```
library(BiocManager)
library(clusterProfiler)
library(AnnotationDbi)
library(org.Tcastaneum.eg.db)
library(stringr)
library(writexl)
library(tidyverse)
library(readxl)
library(ggplot2)

# Prepare data ------------------------------------------------------------

# load in different DESeq2 results: 3h 24h, 5d, 5de, subsequently
a <- read_xlsx("Additional_file_2.xlsx", sheet =5)

a$SYMBOL = mapIds(org.Tcastaneum.eg.db,
               key = a$gene_name,
               column = "SYMBOL",
               keytype = "SYMBOL")

DEG <- subset(a, (log2FoldChange > 0.5 & padj < 0.05) | (log2FoldChange < -0.5 & padj < 0.05), select = SYMBOL)

# define background genes
allg <- a$SYMBOL

# clusterProfiler for over-representation analysis ------------------------

ego <- enrichGO(gene          = DEG$SYMBOL,
                universe      = allg,
                OrgDb         = org.Tcastaneum.eg.db,
                ont           = "ALL",
                pAdjustMethod = "BH",
                pvalueCutoff  = 0.05,
                qvalueCutoff  = 0.05,
                readable      = TRUE,
                keyType = "SYMBOL")

ego1 <- simplify(ego, cutoff=0.7, by="p.adjust", select_fun=min)

b <- data.frame(ego1)

# separate IDs
b1 <- b %>%
  separate_rows(geneID, sep = "/")

# get log2Fcs for IDs
conditions_a <- a[,c(10,34)]

# join the results for respective IDs
b1 <- b1 %>%
  left_join(conditions_a, by = c("geneID" = "SYMBOL"))

# how many up- and how many downregulated, store in b
result_b1 <- b1 %>%
  group_by(ID) %>%
  summarize(up = sum(log2FoldChange > 0) / n() * 100)

result_b2 <- b1 %>%
  group_by(ID) %>%
  summarize(upnum = paste(sum(log2FoldChange > 0), n(), sep = "/"))

result_b3 <- b1 %>%
  group_by(ID) %>%
  summarize(downnum = paste(sum(log2FoldChange < 0), n(), sep = "/"))

b <- b %>%
  left_join(result_b1, by = "ID")%>%
  left_join(result_b2, by = "ID")%>%
  left_join(result_b3, by = "ID")

# save as excel files subsequently for all timepoints
write_xlsx(b, path = "DEGs_3h_slim.xlsx")

# Figure 5G ---------------------------------------------------------------

a <- read_xlsx("DEGs_3h_slim.xlsx")
a <- a[!duplicated(a$geneID), ]

split_gene <- strsplit(a$GeneRatio, "/")

Ratio_gene <- sapply(split_gene, function(x) {
  numerator <- as.numeric(x[1])
  denominator <- as.numeric(x[2])
  result <- numerator / denominator
  return(result)
})

split_global <- strsplit(a$BgRatio, "/")

Ratio_global <- sapply(split_global, function(x) {
  numerator <- as.numeric(x[1])
  denominator <- as.numeric(x[2])
  result <- numerator / denominator
  return(result)
})

Ratio_final <- Ratio_gene / Ratio_global

a$Ratio_f <- Ratio_final

### barplot without subsetting a for different categories
a$up <- as.numeric(a$up)
orderont <- c("BP", "MF", "CC")
a$ONTOLOGY <- factor(a$ONTOLOGY, levels = orderont)
a$Description <- factor(a$Description, levels = unique(a$Description))

colors <- scale_fill_gradient2(low = "#377eb8", mid = "white", high = "#e41a1c", midpoint = 50, limits = c(0, 100), breaks =c(0,50,100))

plot1 <- ggplot(a, aes(x = Count, y = Description, fill = up)) +
  geom_bar(stat = "identity", color ="black") +
  geom_hline(yintercept = 5.5)+
  geom_hline(yintercept = 7.5)+
  colors +
  labs(title = "", y = "", x = "Gene count", fill = "Up-regulated DEGs (%)") +
  theme_bw() + 
  theme(text = element_text(size = 9),
        legend.position = "bottom",
        legend.margin = margin(t = 0.05, unit = "cm"),
        legend.spacing = unit(1, "cm"),
        legend.justification = c(1.2, 0),
        legend.title = element_text(size = 8),
        legend.text = element_text(size = 8, margin = margin(t = 8)),
        legend.key.height = unit(0.3, "cm"),
        legend.key.width = unit(0.3, "cm"),
        theme(text = element_text(family = "Arial")))+
  annotate("text", x = 19, y = 9.5, label = "MF", size = 2.5)+
  annotate("text", x = 19, y = 7, label = "CC", size = 2.5)+
  annotate("text", x = 19, y = 4.5, label = "BP", size = 2.5)

output_file <- "Fig5G.tif"
width_pixels <- 1150  
height_pixels <- 700 

tiff(filename = output_file, width = width_pixels, height = height_pixels, res = 300, units = "px")

print(plot1)
dev.off()

# Figure 6G ---------------------------------------------------------------

b <- read_xlsx("DEGs_24h_slim.xlsx")
b <- b[!duplicated(b$geneID), ]

split_gene <- strsplit(b$GeneRatio, "/")

Ratio_gene <- sapply(split_gene, function(x) {
  numerator <- as.numeric(x[1])
  denominator <- as.numeric(x[2])
  result <- numerator / denominator
  return(result)
})

split_global <- strsplit(b$BgRatio, "/")

Ratio_global <- sapply(split_global, function(x) {
  numerator <- as.numeric(x[1])
  denominator <- as.numeric(x[2])
  result <- numerator / denominator
  return(result)
})

Ratio_final <- Ratio_gene / Ratio_global

b$Ratio_f <- Ratio_final

### barplot without subsetting a for different categories
b$up <- as.numeric(b$up)
orderont <- c("BP", "MF", "CC")
b$ONTOLOGY <- factor(b$ONTOLOGY, levels = orderont)
b$Description <- factor(b$Description, levels = unique(b$Description))

colors <- scale_fill_gradient2(low = "#377eb8", mid = "white", high = "#e41a1c", midpoint = 50, limits = c(0, 100), breaks =c(0,50,100))

plot2 <- ggplot(b, aes(x = Count, y = Description, fill = up)) +
  geom_bar(stat = "identity", color ="black") +
  geom_hline(yintercept = 21.5)+
  geom_hline(yintercept = 11.5)+
  colors +
  labs(title = "", y = "", x = "Gene count", fill = "Up-regulated DEGs (%)") +
  theme_bw() + 
  theme(text = element_text(size = 9),
        legend.position = "bottom",
        legend.margin = margin(t = 0.05, unit = "cm"),
        legend.spacing = unit(1, "cm"),
        legend.justification = c(0.5, 0),
        legend.title = element_text(size = 8),
        legend.text = element_text(size = 8, margin = margin(t = 8)),
        legend.key.height = unit(0.3, "cm"),
        legend.key.width = unit(0.3, "cm"))+
  annotate("text", x = 55, y = 34.5, label = "MF", size = 2.5)+
  annotate("text", x = 55, y = 20.5, label = "CC", size = 2.5)+
  annotate("text", x = 55, y = 10.5, label = "BP", size = 2.5)

output_file <- "Fig6G.tif"
width_pixels <- 1350  
height_pixels <- 1450 

tiff(filename = output_file, width = width_pixels, height = height_pixels, res = 300, units = "px")

print(plot2)
dev.off()

# Figure 7G ---------------------------------------------------------------

c <- read_xlsx("DEGs_5d_slim.xlsx")
c <- c[!duplicated(c$geneID), ]

split_gene <- strsplit(c$GeneRatio, "/")

Ratio_gene <- sapply(split_gene, function(x) {
  numerator <- as.numeric(x[1])
  denominator <- as.numeric(x[2])
  result <- numerator / denominator
  return(result)
})

split_global <- strsplit(c$BgRatio, "/")

Ratio_global <- sapply(split_global, function(x) {
  numerator <- as.numeric(x[1])
  denominator <- as.numeric(x[2])
  result <- numerator / denominator
  return(result)
})

Ratio_final <- Ratio_gene / Ratio_global

c$Ratio_f <- Ratio_final

### barplot without subsetting a for different categories
c$up <- as.numeric(c$up)
orderont <- c("BP", "CC")
c$ONTOLOGY <- factor(c$ONTOLOGY, levels = orderont)
c$Description <- factor(c$Description, levels = unique(c$Description))


colors <- scale_fill_gradient2(low = "#377eb8", mid = "white", high = "#e41a1c", midpoint = 50, limits = c(0, 100), breaks =c(0,50,100))

plot3 <- ggplot(c, aes(x = Count, y = Description, fill = up)) +
  geom_bar(stat = "identity", color ="black") +
  geom_hline(yintercept = 3.5)+
  colors +
  labs(title = "", y = "", x = "Gene count", fill = "Up-regulated DEGs (%)") +
  theme_bw() + 
  theme(text = element_text(size = 9),
        legend.position = "bottom",
        legend.margin = margin(t = 0.05, unit = "cm"),
        legend.spacing = unit(1, "cm"),
        legend.justification = c(0.8, 0),
        legend.title = element_text(size = 8),
        legend.text = element_text(size = 8, margin = margin(t = 8)),
        legend.key.height = unit(0.3, "cm"),
        legend.key.width = unit(0.3, "cm"))+
  annotate("text", x = 6.5, y = 5.5, label = "CC", size = 2.5)+
  annotate("text", x = 6.5, y = 2.5, label = "BP", size = 2.5)

output_file <- "Fig7G.tif"
width_pixels <- 1500  
height_pixels <- 600 

tiff(filename = output_file, width = width_pixels, height = height_pixels, res = 300, units = "px")

print(plot3)
dev.off()

# Figure 8G ---------------------------------------------------------------

d <- read_xlsx("DEGs_5d_C_slim.xlsx")
d <- d[!duplicated(d$geneID), ]

split_gene <- strsplit(d$GeneRatio, "/")

Ratio_gene <- sapply(split_gene, function(x) {
  numerator <- as.numeric(x[1])
  denominator <- as.numeric(x[2])
  result <- numerator / denominator
  return(result)
})

split_global <- strsplit(d$BgRatio, "/")

Ratio_global <- sapply(split_global, function(x) {
  numerator <- as.numeric(x[1])
  denominator <- as.numeric(x[2])
  result <- numerator / denominator
  return(result)
})

Ratio_final <- Ratio_gene / Ratio_global

d$Ratio_f <- Ratio_final

### barplot without subsetting a for different categories
d$up <- as.numeric(d$up)
orderont <- c("BP", "CC", "MF")
d$ONTOLOGY <- factor(d$ONTOLOGY, levels = orderont)
d$Description <- factor(d$Description, levels = unique(d$Description))


colors <- scale_fill_gradient2(low = "#377eb8", mid = "white", high = "#e41a1c", midpoint = 50, limits = c(0, 100), breaks =c(0,50,100))

plot4 <- ggplot(d, aes(x = Count, y = Description, fill = up)) +
  geom_bar(stat = "identity", color ="black") +
  geom_hline(yintercept = 3.5)+
  geom_hline(yintercept = 5.5)+
  colors +
  labs(title = "", y = "", x = "Gene count", fill = "Up-regulated DEGs (%)") +
  scale_x_continuous(breaks = c(0,2,4,6,8,10,12), labels = c(0,2,4,6,8,10,12))+
  theme_bw() + 
  theme(text = element_text(size = 9),
        legend.position = "bottom",
        legend.margin = margin(t = 0.05, unit = "cm"),
        legend.spacing = unit(1, "cm"),
        legend.justification = c(0.8, 0),
        legend.title = element_text(size = 8),
        legend.text = element_text(size = 8, margin = margin(t = 8)),
        legend.key.height = unit(0.3, "cm"),
        legend.key.width = unit(0.3, "cm"))+
  annotate("text", x = 10.5, y = 10.5, label = "MF", size = 2.5)+
  annotate("text", x = 10.5, y = 4.5, label = "CC", size = 2.5)+
  annotate("text", x = 10.5, y = 2.5, label = "BP", size = 2.5)

output_file <- "Fig8G.tif"
width_pixels <- 1500  
height_pixels <- 800 

tiff(filename = output_file, width = width_pixels, height = height_pixels, res = 300, units = "px")

print(plot4)
dev.off()
```

## Fig 9: Proteomics results

The first part will create the full proteomics table. In the main
text, only an exemplary selection of protein groups was represented and
visualized in Fig 9.

```
library(limma) 
library(imputeLCMD)
library(dplyr)
library(tidyverse)
library(ggrepel)
library(writexl)

## read in protein groups and associate with identifiers
sample_assignment <- read.delim("sample_assignment.txt")

proteins <- read.delim("proteingroups.txt")

## remove potential contaminants
proteins <- proteins[proteins$Reverse!="+",]

proteins <- proteins[proteins$Potential.contaminant!="+",]

## subset for log2 transformed LFQ values and set -Inf to NAs

LFQs <- log2(proteins[,grep("LFQ", colnames(proteins))])

LFQs[LFQs=="-Inf"] <- NA

# add coloumn and rownames
colnames(LFQs) <- sample_assignment$sample
rownames(LFQs) <- proteins$Fasta.headers
LFQs$`Fasta headers` <- rownames(LFQs)
LFQs <- LFQs %>%
  select(starts_with("BttCry"), starts_with("Btt 2023"), `Fasta headers`)

#select for <= 1 NAs
selected_Btt <- rowSums(is.na(LFQs[, grepl("^Btt 2023", names(LFQs))])) < 2
selected_Bttm <- rowSums(is.na(LFQs[, grepl("^BttCry-", names(LFQs))])) < 2

# combine lists
selected <- selected_Btt | selected_Bttm

# subset LFQs based on subseted list
LFQs2 <- LFQs[selected, ]

#imputation
set.seed(48149)

LFQs2[,grep("Btt 2023", colnames(LFQs2))] <- imputeLCMD::impute.QRILC(LFQs2[,grep("Btt 2023", colnames(LFQs2))])[[1]]
LFQs2[,grep("BttCry-", colnames(LFQs2))] <- imputeLCMD::impute.QRILC(LFQs2[,grep("BttCry-", colnames(LFQs2))])[[1]]

#limma without median normailization
limma <- eBayes(lmFit(LFQs2[,grep("Btt 2023", colnames(LFQs2))] - LFQs2[,grep("BttCry- ", colnames(LFQs2))]))

volcano_BttCry <- (topTable(limma, number=Inf, adjust="BH", sort.by="none"))
volcano_BttCry <- rownames_to_column(volcano_BttCry, var = "Fasta headers")

# save results from raw data and imputed data
result <- merge(LFQs, volcano_BttCry, by = 'Fasta headers', all.x = TRUE)
result <- result %>%
  filter(`Fasta headers` %in% as.list(volcano_BttCry$`Fasta headers`))
selected_columns <- c("BttCry-  1", "BttCry-  2", "BttCry-  3", "BttCry-  4", "Btt 2023-1", "Btt 2023-2","Btt 2023-3","Btt 2023-4")
new_names <- c("Log2(LFQ C-1)", "Log2(LFQ C-2)", "Log2(LFQ C-3)", "Log2(LFQ C-4)","Log2(LFQ P-1)","Log2(LFQ P-2)","Log2(LFQ P-3)","Log2(LFQ P-4)")
names(result)[names(result) %in% selected_columns] <- new_names

result2 <- merge(result, LFQs2, by = 'Fasta headers', all.x = TRUE)
selected_columns <- c("BttCry-  1", "BttCry-  2", "BttCry-  3", "BttCry-  4", "Btt 2023-1", "Btt 2023-2","Btt 2023-3","Btt 2023-4")
new_names <- c("Imputed C-1", "Imputed C-2", "Imputed C-3", "Imputed C-4","Imputed P-1","Imputed P-2","Imputed P-3","Imputed P-4")
names(result2)[names(result2) %in% selected_columns] <- new_names

result2 <- result2[, c(1:9, 16:23, 10:15)]

write_xlsx(result2, "LFQs.xlsx")


# Figure 9 ----------------------------------------------------------------

library(readxl)
library(ggplot2)
library(ggpubr)
library(stringr)
library(tidyverse)

a <- read_xlsx("Additional_file_2.xlsx", sheet = 15)

# List of IDs to match
ids <- c("BTT_61180", "BTT_61250", "BTT_61290", "UPI0006555EC9", "BTT_38790")

# Create a pattern that matches any of these IDs
pattern <- paste0("^(", paste(ids, collapse = "|"), ")")

# Subset rows based on the pattern
a1 <- a[grepl(pattern, a$`Fasta headers`), ]
a1 <- a1[, c(1, 10:17)]

colnames(a1)[colnames(a1) == "Fasta headers"] <- "ID"

a1 <- a1 %>%
  mutate(Description = case_when(
    ID == "UPI0006555EC9 status=active" ~ "Unknown",
    ID == "BTT_38790 Chitinase D" ~ "ChiD",
    ID == "BTT_61180 Pesticidal crystal protein Cry3Aa" ~ "Cry3Aa",
    ID == "BTT_61250 Pesticidal crystal protein Cry15Aa" ~ "Cry15Aa",
    ID == "BTT_61290 Sphingomyelinase C" ~ "SphC",
    TRUE ~ "Other"
  ))

a1 <- a1[,2:10]

a2 <- gather(a1, key ="Sample", value = "LFQ", -Description)

a2 <- a2 %>%
  mutate(Treatment = case_when(
    Sample %in% c("Imputed C-1", "Imputed C-2", "Imputed C-3", "Imputed C-4") ~ "control",
    Sample %in% c("Imputed P-1", "Imputed P-2", "Imputed P-3", "Imputed P-4") ~ "priming",
    TRUE ~ "Other"
  ))

a2 <- a2 %>% group_by(Treatment, Description) %>% mutate(
  mean_value = mean(LFQ, na.rm = TRUE),
  sd_value = sd(LFQ, na.rm = TRUE),
  n = n(), # Number of observations
  se = sd_value / sqrt(n) # Standard error
)

a2 <- a2[c(1:5, 21:25), c(1, 3:5, 8)]

a2$Description <- factor(a2$Description, levels = c("Cry15Aa", "Unknown", "Cry3Aa","SphC","ChiD"))

plot1 <- ggplot(a2, aes(x = Treatment, y = mean_value, color = Treatment)) +
  geom_point(size = 3) + 
  scale_color_manual(values = c("priming" = "darkolivegreen3", "control" = "darkolivegreen4"))+
  geom_errorbar(aes(ymin = mean_value - se, ymax = mean_value + se), width = 0.5) +
  labs(x = "", y = "log2 LFQ values", color = "Supernatant:") + 
  facet_wrap(~ Description, ncol = 5, nrow = 1) +
  annotate("segment", x = 1, xend = 2, y = 33.5, yend = 33.5, color = "black", size = 0.5)+
  geom_text(
    data = subset(a2, Description == "Cry15Aa"), 
    aes(x = 1.5, y = 34, label = "**"), 
    size = 2.5, 
    color = "black"
  ) +
  geom_text(
    data = subset(a2, Description == "Unknown"), 
    aes(x = 1.5, y = 34, label = "**"), 
    size = 2.5, 
    color = "black"
  ) +
  geom_text(
    data = subset(a2, Description == "Cry3Aa"), 
    aes(x = 1.5, y = 34, label = "*"), 
    size = 2.5, 
    color = "black"
  ) +
  geom_text(
    data = subset(a2, Description == "SphC"), 
    aes(x = 1.5, y = 34, label = "*"), 
    size = 2.5, 
    color = "black"
  ) +
  geom_text(
    data = subset(a2, Description == "ChiD"), 
    aes(x = 1.5, y = 34, label = "0.77"), 
    size = 2.5, 
    color = "black"
  ) +
  theme_bw() +
  theme(
    text = element_text(size = 10),
    legend.position = "bottom",  
    legend.direction = "horizontal",
    axis.text.x = element_blank(),
    legend.margin = margin(t = -15)
  )
plot1

output_file <- "Fig9.tif"
width_pixels <- 1000  
height_pixels <- 900 

tiff(filename = output_file, width = width_pixels, height = height_pixels, res = 300, units = "px")

print(plot1)
dev.off()
```

# Supplementary information

## Additional file 1: Fig S1. Survival for EM and RNA-seq experiments

```
library (survival)                                      
library(ggplot2)    
library(survminer)  
library(coxme)  
library(readxl)
library(dplyr)
library(lmtest)
library(patchwork)

a <- read_xlsx("Additional_file_2.xlsx", sheet = 2)
a$Treatment<-as.factor(a$Treatment) 
a$Experiment<-as.factor(a$Experiment)   

a1 <- subset(a, Experiment ==1)
a2 <- subset(a, Experiment ==3)

# Additional file 1: Fig S1A ---------------------------------------------------------

survs <- function(dat2) {
  names(dat2) <- make.names(names(dat2))  
  S1 <- survfit(Surv(Day_death, Death) ~ Treatment, data = dat2)
  custom_colors <- c("darkolivegreen4", "black", "darkolivegreen3")
  tit <- dat2$...5[1]
  custom_theme = theme_minimal() + theme(panel.border = element_rect(color = "black", fill = NA), axis.title.y = element_text(margin = margin(r = 10)),  # Increase distance of y-axis label from plot
                                         axis.title.x = element_text(margin = margin(t = 6)), axis.text.x = element_text(size = 9),  
                                         axis.text.y = element_text(size = 9),
                                         theme(text = element_text(family = "Arial")))
  ggsurvplot(S1, data = dat2, legend.title = "Survival", xlab = "Time / days", legend.labs = c("control", "medium", "primed"), size = 1, fontsize = 9,
             palette = custom_colors, font.x = 9, font.y = 9, font.legend = 9, title = tit, ggtheme = custom_theme)
}

surv1 <- survs(a1)
surv2 <- survs(a2)

## patchwork
plot1 <- (surv2$plot / surv1$plot) / guide_area() +
  plot_layout(guides = 'collect', axis_titles = "collect")

output_file <- "Additional_file_1_FigS1A.tif"
width_pixels <- 1050  
height_pixels <- 1000 

tiff(filename = output_file, width = width_pixels, height = height_pixels, res = 300, units = "px")

print(plot1)
dev.off()

# Additional file 1: FigS1B --------------------------------------------------------------

m1 = coxph(Surv(Day_death, Death) ~ Treatment, data = a1)
cox.zph(m1)

m2 = coxph(Surv(Day_death, Death) ~ Treatment , data = a2)
cox.zph(m2)

hazard <- function(dat) {
  dat$Treatment = relevel(dat$Treatment, ref = "Med-Btt")
  p1 = coxph(Surv(Day_death, Death) ~ Treatment, data = dat)
  selected_treatments <- c("C-Btt", "P-Btt")
  selected_data <- dat[dat$Treatment %in% selected_treatments, ]
  coef_exp <- exp(coef(p1)) #extract the exponentiated coefficients
  conf_intervals <- confint(p1)
  conf_intervals_exp <- exp(conf_intervals)
  plot_data <- data.frame(
    Treatment = c("control", "primed"),
    exp_coef = coef_exp,
    conf_lower = conf_intervals_exp[, 1],
    conf_upper = conf_intervals_exp[, 2]
  )
  plot_data$Treatment <- factor(plot_data$Treatment, levels = c("primed", "control"))
  tit <- dat$...5[1]
  ggplot(plot_data, aes(x = exp_coef, y = Treatment, color = Treatment)) +
    geom_point(aes(color = Treatment), size = 2) +
    geom_errorbarh(aes(xmin = conf_lower, xmax = conf_upper, color = Treatment), height = 0.05) +
    scale_color_manual(values = c("darkolivegreen3", "darkolivegreen4"))+
    geom_vline(xintercept = 1, linetype = "dashed", color = "gray40") +
    scale_x_log10(limits = c(0.1, 3)) +  
    ggtitle(tit)+
    xlab("Estimates") +
    ylab("Treatment") +
    theme_minimal() +
    theme(text = element_text(size = 9), axis.title.y = element_text(margin = margin(r = 10)),  # Increase distance of y-axis label from plot
          axis.title.x = element_text(margin = margin(t = 10)),
          panel.grid.major.y = element_blank(),  
          panel.grid.minor = element_blank(),  
          panel.grid.major.x = element_line(color = "gray90"),
          panel.border = element_rect(color = "black", fill = NA),
          legend.position = "none",
          plot.title = element_text(size = 9))
}

forest_plot1 <- hazard(a1)
forest_plot2 <- hazard(a2)

## patchwork
plot2 <- (forest_plot2 / forest_plot1) / guide_area() +
  plot_layout(guides = 'collect', axis_titles = "collect", axes = "collect") 

output_file <- "Additional_file_1_S1B.tif"
width_pixels <- 800  
height_pixels <- 800 

tiff(filename = output_file, width = width_pixels, height = height_pixels, res = 300, units = "px")

print(plot2)
dev.off()
```

## Additional file 1: Fig S2. WGCNA dendogram

```
library(WGCNA)

bwnet1 <- readr::read_rds("WGCNA_priming.RDS")

mergedColors = labels2colors(bwnet1$colors)
unmergedColors = labels2colors(bwnet1$unmergedColors)
# Plot the dendrogram and the module colors underneath

plotDendroAndColors(bwnet1$dendrograms[[1]],
  cbind(mergedColors[bwnet1$blockGenes[[1]]], unmergedColors[bwnet1$blockGenes[[1]]]),
  c("Merged", "Unmerged"),
  dendroLabels = FALSE,
  hang = 0.03,
  addGuide = TRUE,
  guideHang = 0.05)
```

## Additional file 1: FigS3. WGCNA pearson heatmap

```
library(WGCNA)
library(CorLevelPlot)
library(tidyverse)
library(readxl)
library(ComplexHeatmap)

a <- read_xlsx("Additional_file_2.xlsx", sheet = 4)
a <- a[, 1:25]
a <- a %>%
  filter(rowSums(.[, 2:25]) >= 24)
a <- column_to_rownames(a, var = "gene_id")

colData <- read_xlsx("Additional_file_2.xlsx", sheet = 9)
colData <- as.data.frame(colData)
rownames(colData) <- colData$...1
colData <- colData[, -1]
colData$Group <- as.factor(colData$Group)

bwnet1 <- readr::read_rds("WGCNA_priming.RDS")
module_eigengenes <- bwnet1$MEs

# Additional file 1: FigS3 ----------------------------------------------------

groups.model <- colData %>%
  mutate("P-3h" = ifelse(grepl("P_3h",Group),1,0))%>%
  mutate("C-3h" = ifelse(grepl("C_3h",Group),1,0))%>%
  mutate("P-24h" = ifelse(grepl("P_24h",Group),1,0))%>%
  mutate("C-24h" = ifelse(grepl("C_24h",Group),1,0))%>%
  mutate("P-5d-E" = ifelse(grepl("P_5d_C",Group),1,0))%>%
  mutate("C-5d-E" = ifelse(grepl("C_5d_C",Group),1,0))%>%
  mutate("P-5d" = ifelse(grepl("P_5d$",Group),1,0))%>%
  mutate("C-5d" = ifelse(grepl("C_5d$",Group),1,0))
groups.model <- groups.model[,6:13]

neworder <- c("C-3h", "P-3h", "C-24h", "P-24h", "C-5d", "P-5d","C-5d-E", "P-5d-E")
groups.model <- groups.model[, order(match(names(groups.model), neworder))]

nsamples <- nrow(bwnet1$MEs)
ngenes <- 10814

MEorder <- orderMEs(module_eigengenes[,1:25])
mod.group.cor <- cor(MEorder,groups.model,use = "p")
mod.group.cor.pval <- corPvalueStudent(mod.group.cor, nsamples)

correl <- as.data.frame(mod.group.cor)

correl <- as.matrix(correl)

color_func <- circlize::colorRamp2(
  c(-1, 0, 1),
  c("#377eb8", "white", "#e41a1c"))

pvalues <- mod.group.cor.pval
asterisks <- ifelse(pvalues < 0.001, "***", ifelse(pvalues < 0.01, "**", ifelse(pvalues < 0.05, "*", "")))

column_group <- c("3 hours", "3 hours", "24 hours", "24 hours", "5 days", "5 days", "5 days +Btt", "5 days +Btt")
neworder2 <- c("3 hours", "24 hours", "5 days", "5 days +Btt")
column_group2 <- factor(column_group, levels = neworder2)

correl_re <- correl[, order(match(colnames(correl), neworder))]

colnames(correl_re) <- c("control", "primed", "control", "primed", "control", "primed", "control", "primed")

# plot
plot1 <- ComplexHeatmap::Heatmap(
  correl_re,
  col = color_func,
  cluster_columns = F,
  show_row_names = T,
  row_names_gp = grid::gpar(fontsize = 8),
  row_names_side = "left",
  show_column_names = TRUE,
  column_names_gp = grid::gpar(fontsize = 8),
  use_raster = FALSE,  # Set to TRUE for high-resolution output
  cluster_rows = T,  # Rows will not be clustered,
  heatmap_legend_param = list(
    at = c(-1, 0, 1, 0.5, -0.5),
    legend_height = unit(3, "cm"),
    grid_width = unit(0.5, "cm"),
    title = "",
    labels_gp = gpar(fontsize = 8)),
  cell_fun = function(j, i, x, y, width, height, fill) {
    grid.rect(x, y, width = width, height = height, gp = gpar(col = "black", fill = fill))
    grid.text(
      sprintf("%.2f%s", correl[i, j], asterisks[i, j]),
      x, y, gp = gpar(fontsize = 8))
  },
  column_names_rot = 45,
  column_names_centered = F,
  column_split = column_group2,
  column_title_gp = gpar(fontsize = 8)
)

output_file <- "Additional_file_1_S3.tif"
width_pixels <- 1400  
height_pixels <- 1000 

tiff(filename = output_file, width = width_pixels, height = height_pixels, res = 300, units = "px")

print(plot1)
dev.off()
```

## Additional file 1: Fig S4. Proteomics volcano plot

```
library(limma) 
library(imputeLCMD)
library(dplyr)
library(tidyverse)
library(ggrepel)
library(writexl)

## read in metadata and data
sample_assignment <- read.delim("sample_assignment.txt")

proteins <- read.delim("proteingroups.txt")

## remove potential contaminants
proteins <- proteins[proteins$Reverse!="+",]

proteins <- proteins[proteins$Potential.contaminant!="+",]

## subset for log2 transformed LFQ values and set -Inf to NAs

LFQs <- log2(proteins[,grep("LFQ", colnames(proteins))])

LFQs[LFQs=="-Inf"] <- NA

# add coloumn and rownames
colnames(LFQs) <- sample_assignment$sample
rownames(LFQs) <- proteins$Fasta.headers
LFQs$`Fasta headers` <- rownames(LFQs)
LFQs <- LFQs %>%
  select(starts_with("BttCry"), starts_with("Btt 2023"), `Fasta headers`)

#select for <= 1 NAs
selected_Btt <- rowSums(is.na(LFQs[, grepl("^Btt 2023", names(LFQs))])) < 2
selected_Bttm <- rowSums(is.na(LFQs[, grepl("^BttCry-", names(LFQs))])) < 2

# combine lists
selected <- selected_Btt | selected_Bttm

# subset LFQs based on subseted list
LFQs2 <- LFQs[selected, ]

# Additional file 1: FigS4A ----------------------------------------------------------

#limma
limma <- eBayes(lmFit(LFQs2[,grep("Btt 2023", colnames(LFQs2))] - LFQs2[,grep("BttCry- ", colnames(LFQs2))]))

volcano_BttCry1 <- (topTable(limma, number=Inf, adjust="BH", sort.by="none"))
volcano_BttCry1 <- rownames_to_column(volcano_BttCry1, var = "Fasta headers")

# select points to highlight
volcano_BttCry1 <- volcano_BttCry1 %>%
  mutate(color_group = case_when(
    volcano_BttCry1$adj.P.Val < 0.05 & logFC > 1 ~ "Upregulated",
    volcano_BttCry1$adj.P.Val < 0.05 & logFC < -1 ~ "Downregulated",
    TRUE ~ "Other"
  ))

selected_points <- subset(volcano_BttCry1, adj.P.Val < 0.06)
selected_points$custom_label <- "Sphingomyelinase C"

# volcano plot without imputation
plot1 <- ggplot(data = volcano_BttCry1, aes(logFC, -log10(adj.P.Val)))+
  geom_point(aes(color = color_group), size = 2)+
  scale_y_continuous(limits = c(0,3), breaks = c(0,0.5, 1, 1.5, 2, 2.5,3))+
  scale_x_continuous(limits = c(-8, 15))+
  xlab("log2FC")+
  geom_vline(xintercept = 1) +
  geom_vline(xintercept = -1) +
  geom_hline(yintercept = 1.301)+ 
  scale_color_manual(values = c("black", "darkolivegreen3"))+
  theme_bw()+
  theme(text = element_text(size = 9))+
  theme(legend.position = "none") +
  geom_text_repel(data = selected_points, aes(label = custom_label), 
                  box.padding = 0.4, point.padding = 0.1, size = 3) 

output_file <- "Additional_file_1_FigS4A.tif"
width_pixels <- 750  
height_pixels <- 750 

tiff(filename = output_file, width = width_pixels, height = height_pixels, res = 300, units = "px")

print(plot1)
dev.off()

# Additional file 1: FigS4B ----------------------------------------------------------

#imputation
set.seed(48149)

LFQs2[,grep("Btt 2023", colnames(LFQs2))] <- imputeLCMD::impute.QRILC(LFQs2[,grep("Btt 2023", colnames(LFQs2))])[[1]]
LFQs2[,grep("BttCry-", colnames(LFQs2))] <- imputeLCMD::impute.QRILC(LFQs2[,grep("BttCry-", colnames(LFQs2))])[[1]]

# limma
limma <- eBayes(lmFit(LFQs2[,grep("Btt 2023", colnames(LFQs2))] - LFQs2[,grep("BttCry- ", colnames(LFQs2))]))

volcano_BttCry <- (topTable(limma, number=Inf, adjust="BH", sort.by="none"))
volcano_BttCry <- rownames_to_column(volcano_BttCry, var = "Fasta headers")

# select points to highlight
volcano_BttCry <- volcano_BttCry %>%
  mutate(color_group = case_when(
    volcano_BttCry$adj.P.Val < 0.05 & logFC > 1 ~ "Upregulated",
    volcano_BttCry$adj.P.Val < 0.05 & logFC < -1 ~ "Downregulated",
    TRUE ~ "Other"
  ))

selected_points <- subset(volcano_BttCry, adj.P.Val < 0.05)
selected_points$custom_label <- c("Cry3Aa", "Cry15Aa", "Sphingomyelinase C", "Unknown")

# volcano plot with imputation
plot2 <- ggplot(data = volcano_BttCry, aes(logFC, -log10(adj.P.Val)))+
  geom_point(aes(color = color_group), size = 2)+
  scale_y_continuous(limits = c(0,3), breaks = c(0,0.5, 1, 1.5, 2, 2.5,3))+
  scale_x_continuous(limits = c(-8, 15))+
  xlab("log2FC")+
  geom_vline(xintercept = 1) +
  geom_vline(xintercept = -1) +
  geom_hline(yintercept = 1.301)+ 
  scale_color_manual(values = c("black", "darkolivegreen3"))+
  theme_bw()+
  theme(text = element_text(size = 9))+
  theme(legend.position = "none") +
  geom_text_repel(data = selected_points, aes(label = custom_label), 
                  box.padding = 0.3, point.padding = 0.05, size = 3) 

output_file <- "Additional_file_1_S4B.tif"
width_pixels <- 750  
height_pixels <- 750 

tiff(filename = output_file, width = width_pixels, height = height_pixels, res = 300, units = "px")

print(plot2)
dev.off()
```
